# Discovery of potential ovicidal natural products using metabolomics

**DOI:** 10.1371/journal.pone.0211237

**Published:** 2019-01-25

**Authors:** Dyego Gonçalves Lino Borges, Jessica Teles Echeverria, Tamires Lima de Oliveira, Rafael Pereira Heckler, Mariana Green de Freitas, Geraldo Alves Damasceno-Junior, Carlos Alexandre Carollo, Fernando de Almeida Borges

**Affiliations:** 1 School of Veterinary Medicine and Animal Science, Federal University of Mato Grosso do Sul, Campo Grande, Mato Grosso do Sul, Brazil; 2 Institute of Biosciences, Federal University of Mato Grosso do Sul, Campo Grande, Mato Grosso do Sul, Brazil; 3 School of Pharmaceutical Sciences, Food and Nutrition, Federal University of Mato Grosso do Sul, Campo Grande, Mato Grosso do Sul, Brazil; Instituto Butantan, BRAZIL

## Abstract

Plant extracts are a potential source of new compounds for nematode control and may be an excellent alternative for the control gastrointestinal nematodes that are resistant to conventional anthelmintics. However, research involving natural products is a complex process. The main challenge is the identification of bioactive compounds. Online analytical techniques with universal detectors, such as high-performance liquid chromatography-mass spectrometry (HPLC-MS), together with metabolomics could enable the fast, accurate evaluation of a massive amount of data, constituting a viable option for the identification of active compounds in plant extracts. This study focused on the evaluation of the ovicidal activity of ethanol extracts from 17 plants collected from the Pantanal wetland in the state of Mato Grosso do Sul, Brazil, against eggs of *Haemonchus placei* using the egg hatchability test. The ethanol extracts were obtained using accelerated solvent extraction. The data on ovicidal activity, mass spectrometry and metabolomics were evaluated using HPLC-DAD-MS, partial least squares regression analysis (PLS-DA) and a correlation map (univariate correlation analyses) to detect compounds that have a positive correlation with biological activity. Among the ten metabolites with the best correlation coefficients, six were phenylpropanoids, two were triterpene saponins, one was a brevipolide, and one was a flavonoid. Combinations of metabolites with high ovicidal action were also identified, such as phenylpropanoids combined with the triterpene saponins and the flavonoid, flavonoids combined with iridoid and phenylpropanoids, and saponins combined with phenylpropanoid. The positive correlation between classes of compounds in plants belonging to different genera and biological activity (as previously identified in the literature) reinforces the robustness of the statistical data and demonstrates the efficacy of this method for the selection of bioactive compounds without the need for isolation and reevaluation. The proposed method also enables the determination of synergism among the classes, which would be impracticable using traditional methods. The present investigation demonstrates that the metabolomic technique was efficient at detecting secondary metabolites with ovicidal activity against *H*. *placei*. Thus, the use of metabolomics can be a tool to accelerate and simplify bioprospecting research with plant extracts in veterinary parasitology.

## Introduction

Gastrointestinal nematodiasis (GIN) substantially affects cattle health, particularly in cases of co-infection by infectious and parasitic agents [1]. Among parasitic species of veterinary concern, the hematophagous nematode *Haemonchus placei* is particularly pathogenic to cattle in tropical regions, causing hypoproteinemia, anemia, and anorexia in host animals [2], often with considerable economic losses.

Given the economic impact of GIN on cattle [3,4] and considering the increasing difficulty in controlling these parasitic nematodes with traditional anthelmintic drugs, especially *H*. *placei* [5,6,7], plant-based agents constitute a promising alternative [8,9,10,11]. Plants extracts are a potential source of new compounds for the control of nematodes [12] and the use of plant-based drugs with anthelmintic activity may be an excellent alternative for controlling GIN in ruminants [9, 11]. However, research involving natural products is a complex task. The main challenge is the identification of bioactive compounds in plant extracts [11].

Online analytical techniques with universal detectors, such as high-performance liquid chromatography coupled to mass spectrometry (HPLC-MS), can speed up the discovery of new compounds from plants as well as identify those that have previously been reported in the literature [13]. When combined with metabolomic tools, this strategy could enable the fast, accurate evaluation of a massive amount of data, constituting a viable option for the identification of active compounds in plant extracts [14,15].

Therefore, the present study aimed to evaluate the ovicidal activity of extracts from 17 plants collected from the Pantanal wetland in Brazil against eggs of *H*. *placei* using accelerated solvent extraction, mass spectrometry and metabolomic tools to determine bioactive compounds.

## Materials and methods

The biological samples used in this study (plants and parasite) were registered with the SisGen plataform (code: A63DB37). All activities that were performed with animals respected the ethical principles adopted by the National Council for the Control of Animal Experimentation (NCCAE), and were approved by the Committee on Ethics in the Use of Animals / CEUA / UFMS with Protocol n°. 475/2012.

### 2.1. *Haemonchus placei* isolate

HpIBR1 is an isolate of *H*. *placei* cryopreserved at the Laboratory of Veterinary Parasitology of the Federal University of Mato Grosso do Sul (UFMS). This isolate has been phenotypically characterized as resistant to ivermectin [16].

For maintenance of the isolate, a calf was dewormed for three consecutive days with oral albendazole sulfoxide (Ricobendazole, Ourofino, Brazil) at a concentration of 7.5 mg/kg. The animal was evaluated daily for GIN infection by fecal egg counts [17] and was considered free of helminth infection when 30 days had elapsed with no fecal helminth detected, at which point the animal was orally inoculated with 10,000 third-stage larvae of *H*. *placei*. From the 30^th^ day post-inoculation, fecal samples were collected daily to obtain eggs for use in the *in vitro* tests of the plant extracts.

### 2.2. Plant material

Seventeen plant species (Table 1) were randomly selected from the Corumbá River–Abobral region (São Miguel farm, Estrada Parque, UFMS, Pantanal Field Station and São Bento farm) in the rainy season (December 2011 to March 2012). A voucher specimen of each species was deposited at the CGMS Herbarium of the UFMS.

**Table 1:** Families, species, code, collection sites, parts collected and popular names of plants evaluated for ovicidal activity against *Haemonchus placei*.

Family	Species	Code	Collection site	Part collected	Popular name
**Alismataceae**	*Echinodorus paniculatus* Micheli	*E_panic*	19°34’36”S; 57°1’11”W	AP, FL	Chapéu-de-couro
**Alismataceae**	*Melanthera latifolia*(Gardner) Cabrera	*M_latif*	19°34’36”S; 57°1’11”W	AP, FL	–
**Asteraceae**	*Aspilia latissima* Malme	*A_latis*	19°34’36”S; 57°1’11”W	LE	Fumeiro
**Asteraceae**	*Centratherum punctatum* Cass.	*C_punct*	19°37’5” S; 57°2’4”W	AP, FL	Balaio-de-velho
**Bignoniaceae**	*Handroanthus serratifolius* (Vahl) S.O. Grose	*H_serra*	19°34’36”S; 57° 1’11”W	FL	Ipê-amarelo
**Convolvulaceae**	*Ipomoea chiliantha* Hallier f.	*I_chili*	19°37’5” S; 57°2’4”W	AP	Cipó-de-leite
**Euphorbiaceae**	*Sebastiana hispida* (Mart.) Pax	*S_hispi*	19°34’7”S; 57°1’15”W	AP	Mercúrio
**Hippocrateaceae**	*Hippocratea volubilis* L.	*I_volub*	19°34’7”S; 57°1’15”W	TW, LE	Fava-de-arara
**Lamiaceae**	*Hyptis brevipes* Poit	*H_brevi*	19°34’7”S; 57°1’15”W	WP, FL	Alfavaca-do-mato
**Lamiaceae**	*Hyptis mutabilis* (Rich.) Briq.	*H_mutab*	19°34’7”S; 57°1’15”W	AP, FL	Sambacuité, cheirosa, betônica-brava
**Lauraceae**	*Ocotea diospyrifolia* (Meisn.) Mez	*O_diosp*	19°36’30”S; 57°2’8”W	TW, LE	Canela-preta
**Rubiaceae**	*Dioidia kuntzei* K. Schum.	*D_kuntz*	19°34’7”S; 57°1’15”W	WP, FL	–
**Rubiaceae**	*Tocoynea formosa* (Cham. & Schltdl.) K. Schum.	*T_form*	19°34’7”S; 57°1’15”W	TW, LE	Jenipapinho
**Salicaceae**	*Casearia aculeata* Jacq.	*C_acule*	19°34’7”S; 57°1’15”W	TW, LE, FL	Cruzeiro
**Scrophulareaceae**	*Angelonia hirta* Cham.	*A_hirta*	19°34’7”S; 57°1’15”W	AP, FL, FR	–
**Verbenaceae**	*Lantana canescens* Kunth	*L_canes*	19°37’5” S; 57°2’4”W	AP, FL	Camara, cidreira
**Verbenaceae**	*Vitex cymosa* Bertero ex Spreng.	*V_cymos*	19°34’7”S; 57°1’15”W	TW, LE	Azeitona-do-mato, jaramantaia, tarumã

TW: twigs; LE: leaves; FR: fruit; AP: aerial parts (including leaves, twigs and stems); FL: flowers; RO: roots; WP: whole plant (including roots, leaves and twigs.

### 2.3. Plant extracts

The collected material was initially stabilized and dried in a forced-draft oven at 40 °C. Extraction was performed in an ASE 150 accelerated solvent extraction (Dionex) with ethanol:water (7:3) as the solvent at 100 °C and 1600 psi in a single cycle with a 5-min static time, 60% rinse volume and 50 s purge time. The extracts were concentrated in a rotary evaporator, stored in 15-mL Falcon tubes, labeled, sealed and kept at –20 °C until testing.

### 2.4 Egg hatchability test

Eggs were immediately recovered from fresh fecal samples using the procedure described by Coles et al. [18], adapted by Bizimenyera et al. [19] and further modified as follows: A 500-μL aliquot of water containing approximately 100 eggs was pipetted into each plate well, followed by the addition of 500 μL of either the treatment solution or water (control). The plates were incubated at 27 °C for 24 h. The assays were performed in triplicate for each extract concentration using 24-well cell culture plates.

### 2.5. Ovicidal evaluation

Two egg hatchability tests were used to evaluate the effect of the extracts against *H*. *placei* eggs. In experiment I (screening test), the extracts were weighed, diluted in distilled water using a vortex (IKA) and subsequently evaluated at concentrations of 1, 10, 50, and 100 mg/mL (Table 2). In experiment II, the 13 species that demonstrated efficacy in the screening test were evaluated further: *Angelonia hirta*, *Aspilia latissima*, *Centratherum punctatum*, *Dioidia kuntzei*, *Echinodorus paniculatus*, *Hyptis brevipes*, *Hyptis mutabilis*, *Ipomoea chiliantha*, *Lantana canescens*, *Sebastiana hispida*, *Handroanthus serratifolius*, *Tocoynea formosa* and *Vitex cymosa*. As small amounts of sediment were found in assays with *H*. *serratifolius* and *T*. *formosa*, the EC_90_ and EC_50_ values for these species were obtained from data collected after the removal of the residue. The concentration that led to the lowest hatching rate in experiment I was employed as the high dose, from which nine other dilutions at 1:2 ratios were serially prepared.

**Table 2:** Plant species, extract concentrations screened, hatching rates and *p*-values (water/extract concentration).

Species	Hatching rate (%)					*p*-value
	Water	1 mg/mL	10 mg/mL	50 mg/mL	100 mg/mL	
*Angelonia hirta*	78.4^b^	79.3^b^	58.5^a^	40.3^a^	N.E.	0.0001
*Aspilia latissimi*	73.4^b^	80.1^b^	5.7^a^	0.0^a^	N.E.	<0.0001
*Casearia aculeate*	89.7^a^	88.7^a^	92.7^a^	89.8^a^	N.E.	0.0789
*Centratherum punctatum*	86.5^c^	N.E.	59.5^b^	57.0^ab^	2.3^a^	<0.0001
*Diodia kuntzei*	79.2^c^	58.6^b^	18.5^a^	18.0^a^	N.E.	<0.0001
*Echinodorus paniculatus*	78.0^c^	78.5^bc^	0.6^ab^	21.4^ab^	N.E.	<0.0001
*Hippocratea volubilis*	80.7^b^	84.9^ab^	74.9^a^	83.9^ab^	N.E.	0.0155
*Hyptis brevipes*	76.7^c^	73.7^bc^	57.3^bc^	43.3^ab^	N.E.	0.0199
*Hyptis mutabilis*	81.0^b^	78.9^b^	12.6^a^	8.4^a^	N.E.	<0.0001
*Ipomoea chiliantha*	95.3^b^	N.E.	4.2^a^	0.7^a^	0.6^a^	<0.0001
*Lantana canescens*	83.4^b^	N.E.	0.0^a^	19.3^a^	7.9^a^	0.0024
*Melanthera latifolia*	74.0^a^	86.0^b^	88.0^b^	80.0^ab^	N.E.	0.0023
*Ocotea diospyrifolia*	75.4^a^	89.2^b^	88.9^b^	84.4^ab^	N.E.	0.0143
*Sebastiana hispida*	93.2^b^	N.E.	0.7^a^	2.7^a^	2.9^a^	<0.0001
*Handroanthus serratifolius*	84.0^d^	95.4^d^	40.9^c^	18.5^b^	0.0^a^	<0.0001
*Tocoyena formosa*	81.0^b^	83.9^b^	53.8^a^	72.5^b^	N.E.	0.0001
*Vitex cymosa*	83.0^c^	N.E.	57.4^b^	1.4^a^	0.0^a^	<0.0001

Different letters on the same row indicate significant differences (α = 0.05, one-way ANOVA followed by Bonferroni’s post-hoc test); N.E.: Not Evaluated.

The concentration ranges tested in the experiment II were as follows: *A*. *hirta*, 100–0.19 mg/mL; *A*. *latissima*, 50–0.10 mg/mL; *C*. *punctatum*, 120–0.23 mg/mL; *D*. *kuntzei*, 100–0.19 mg/mL; *E*. *paniculatus*, 100–0.19 mg/mL; *H*. *mutabilis*, 80–0.16 mg/mL; *H*. *brevipes*, 100–0.19 mg/mL; *I*. *chiliantha*, 50–0.10 mg/mL; *L*. *canescens*, 10–0.02 mg/mL; *S*. *hispida*, 50–0.10 mg/mL; *H*. *serratifolius*, 3.3–0.006 mg/mL; *T*. *formosa*, 120–0.23 mg/mL; and *V*. *cymosa*, 50–0.10 mg/mL. The positive control (thiabendazole) was tested in the range of 5 × 10^−4^ to 1 × 10^−6^ mg/mL.

### 2.6. HPLC-DAD-MS analyses

The extracts were analyzed in triplicate by random sampling in an HPLC coupled to a diode array detector (DAD) (Shimadzu) and a mass spectrometer ESI-qTOF microTOF-Q III (Bruker Daltonics). Separation of the compounds was performed by a Kinetex C-18 (2.6 μ, 150 x 2.2 mm, Phenomenex) chromatographic column protected by a pre-column. The mobile phase was ultrapure water (solvent A) and acetonitrile (solvent B), both with 0.1% formic acid (v/v). The following was the gradient elution: 0–2 min 3% B; 2-25min 3–25% B; 25–40 min 25–80% B, followed by column washing and reconditioning (8 minutes). The flow rate was 0.3 mL/min. The column oven was 50 °C and the injection volume was 1 μL. The UV analyses were performed in the wavelength range of 240–800 nm, with the mass spectrometer operating in negative and positive mode (*m/z* 120–1200). The samples used for the quality control of the metabolic analyses consisted of a pool produced by the addition of 50 μL of each sample, which was injected in every six analyses. The pool data were not considered in the statistical analysis. The identification of the compounds was based on mass spectrometry (accurate mass and ion fragmentation pathway) and UV data compared to information reported in the literature. The molecular formula of each compound was determined based on the mass errors within ± 5 ppm and mSigma below 30.

### 2.7. Statistical analysis

In the screening test, hatching rates were expressed as percentages [18]:

Hatchingrate(%)=[numberoflarvae/(numberofeggs+larvae)]×100

The data were subjected to one-way analysis of variance (ANOVA) with hatchability as the single factor, followed by Bonferroni’s post-hoc test (α = 0.05). All treatments were compared to the controls (water) and to each other. Extracts were considered active when at least one of the concentrations led to lower hatching rates than those observed in the controls (*p* < 0.05).

Dose *vs*. response sigmoid curves were constructed by non-linear regression for the active extracts based on 10 concentrations. The concentrations were log-transformed (X = log X) and effectiveness values (for each repetition) were expressed as percentages. The EC_50_ (mean effective concentration) and EC_90_ were calculated using the following equations: *Y = 100 / (1 + 10^((Log EC*_*50*_ –X) * HillSlope)) and log EC_*50*_ = *log EC*_*90*_ –(1 / HillSlope) * log (90 / (100–90)) where Y = Bottom + (Top–Bottom) / (1 + 10^((Log EC_*50*_ –X) * HillSlope)), in which X = concentration log, Y = effectiveness (%), Bottom = minimum effectiveness, Top = maximum effectiveness, and HillSlope = the dose *vs*. response curve slope. EC_90_/EC_50_ ratios were expressed as mg/mL.

Statistical analysis was performed using GraphPad Prism 6.0 software (GraphPad Software, San Diego, CA, USA, http://www.graphpad.com). For each extract concentration, the hatchability inhibition rate was calculated as follows [18]:

Hatchabilityinhibitionrate(%)=[numberofeggs/(numberofeggs+larvae)]×100.

HPLC-DAD-MS analyses were processed in DataAnalysis 4.2 in negative mode, which shows better ionization and a larger number of peaks. Data were aligned using the Metalign software [20], resulting in 1029 entries, which were reduced by MSclust [21]. The entries were regrouped, resulting in 98 reconstituted metabolites from the assembled signals (different ions of the same molecules, such as isotopes, fragments, and adducts). The replicates and pool samples were compared to evaluate the reproducibility of the equipment and processing. The exported data were analyzed with the aid of the MetaboAnalyst 4.0 platform [22], using univariate analysis tools to correlate the active extracts and main compounds of the extracts with Pattern Hunter. In the correlation map between the chemical composition and biological activity determined to apply Pearson’s correlations (significant at p ≤ 0.05), the metabolomic study with the final data referring to EC_90_ was performed using base 1/EC_90_, since lower ECs constitute the best results. Compounds with minimum intensity peaks of 20000 in the mass spectrometric analysis were considered for the correlation map between chemical composition and biological activity.

The plants were grouped based on the results of egg hatchability test (experiment I) and analyzed by discriminated partial least squares regression analysis (PLS-DA). The aim of this analysis, which is based primarily on the chemical composition of the extracts, was to characterize the metabolism of active and inactive extracts. The goodness and robustness of the PLS-DA model were estimated from calculations of R^2^ and Q^2^, respectively. R^2^ is the fraction of variance explained by a component and Q^2^ describes the total fraction predicted by a component. A Q^2^ value > 0.4 characterizes a model as good and Q^2^ > 0.7 characterizes the model as robust [23]. A correlation map was used to detect compounds with a positive correlation to the EC_90_.

## Results

### Experiment I

Thirteen plants exhibited significant activity during the screening test. *Melanthera latifolia*, *Casearia aculeata*, and *Ocotea diospyrifolia* were ineffective during the screening test (Table 2), with respective hatchability inhibition rates of 19.85%, 10.35% and 15.9% for the highest concentration tested. Higher hatching rates (*p* = 0.0023) were found when exposing the eggs to the *M*. *latifolia* (*p* = 0.0023) and *O*. *diospyrifolia* (*p* = 0.0143) extracts at concentrations of 1 to 10 mg/mL compared to exposure to the negative control (water). *Hippocratea volubilis* exhibited activity only at 10 mg/mL, reducing the hatching rate (*p* = 0.0155). However, based on the need for a high dose, we do not consider it to be a promising species.

### 3.2. Experiment II

All 13 extracts evaluated at 10 serial concentrations (*A*. *latissima*, *C*. *punctatum*, *D*. *kuntzei*, *E*. *paniculatus*, *H*. *mutabilis*, *H*. *brevipes*, *I*. *chiliantha*, *L*. *canescens*, *S*. *hispida*, *H*. *serratifolius*, *V*. *cymosa*, *A*. *hirta* and *T*. *formosa*) exhibited dose-dependent behavior, with a gradual increase in effectiveness with the increase in concentration. EC_50_ values ranged from 0.5 to 13.13 mg/mL and EC_90_ values ranged from 1.18 to 174.6 mg/mL (Table 3).

**Table 3:** Plant species, mean effective concentrations inhibiting hatchability of *Haemonchus placei* eggs by 50% (EC_50_) and 90% (EC_90_), confidence intervals, curve slopes (HillSlope) and coefficient of determination (R^2^) obtained from serial evaluations of 10 concentrations.

Species	EC_50_ (mg/mL)	95% CI	HillSlope	R^2^	EC_90_ (mg/mL)	95% CI	HillSlope	R^2^
*Angelonia hirta*	2.61	1.98–3.43	1.08	0.89	6.87	5.34–8.85	4.90	0.95
*Aspilia latissima*	1.28	0.99–1.66	1.56	0.88	2.48	1.83–3.34	7.03	0.98
*Centratherum punctatum*	12.42	10.19–15.13	1.54	0.93	42.07	23.17–76.36	1.99	0.95
*Dioidia kuntzei*	2.60	2.33–2.91	2.92	0.96	4.75	3.90–5.77	4.56	0.96
*Echinodorus paniculatus*	6.43	5.36–7.72	1.30	0.94	21.45	14.36–32.04	2.07	0.97
*Hyptis brevipes*	3.34	3.04–3.66	3.03	0.97	5.71	5.10–6.40	4.70	0.99
*Hyptis mutabilis*	2.43	1.98–2.98	1.59	0.91	5.51	4.69–6.47	4.04	0.98
*Ipomoea chiliantha*	0.54	0.35–0.82	1.03	0.73	1.19	0.19–7.11	15.49	0.98
*Lantana canescens*	0.75	0.46–1.22	1.46	0.43	1.70	0.71–4.09	8.35	0.98
*Sebastiana hispida*	1.88	1.64–2.15	3.86	0.93	2.72	2.13–3.46	7.07	0.98
*Handroanthus serratifolius*	0.83	0.72–0.96	2.88	0.83	1.94	1.25–3.01	3.16	0.88
*Tocoynea formosa*	10.08	5.55–18.18	0.58	0.64	14.52	4.98–42.34	2.99	0.74
*Vitex cymosa*	13.13	6.53–26.38	0.45	0.57	174.60	1.61–18915	1.29	0.78
Thiabendazole	2 × 10^−6^	1.9 × 10^−6^–2.2 × 10^−6^	1.68	0.98	6 × 10^−6^	4 × 10^−6^–9 × 10^−6^	1.26	0.98

Complete inhibition was achieved with extracts from *A*. *hirta* (100 mg/mL), *D*. *kuntzei* (25 mg/mL), *H*. *brevipes* (25 mg/mL), *A*. *latissima* (6.25 mg/mL), *T*. *formosa* (15 mg/mL), *I*. *chiliantha* (6.25 mg/mL), *L*. *canescens* (5 mg/mL) and *H*. *serratifolius* (2.33 mg/mL). Eggs treated with the *S*. *hispida* extract exhibited an amorphous coating and viable larvae inside the eggs at the end of the incubation period, but hatching was low or not observed.

### 3.3. HPLC-DAD-MS analyses

Six classes of secondary metabolites were positively correlated to ovicidal action against *H*. *placei*. The active classes were identified as iridoids (peaks 2, 3, 4, 5, 6, 8 and 9), phenylpropanoids (peaks 7, 12, 18, 20, 23, 25 and 26), hydrolysable tannins (peaks 10 and 11), flavonoids (13, 14, 15, 16, 17, 21, 24 and 30), triterpene saponins (peaks 28, 31, 32, 34, 35, 36 and 37) and brevipolides (peaks 29 and 33) (Table 4).

**Table 4:** Retention time, UV spectrum, mass, molecular formula, chemical class and plants species in which compounds were detected of active peaks with relative intensity of masses higher than 20000 and were positively correlated to EC_90_.

**Peak**	**Ret** **(min)**	**UV**	**[M-H]-**	**Molecular formula**	**MS/MS**	**Class**	**Compounds**	**Plants**
**1**	1.1	---	665.2114	---	---	Unknown	Unknown	*H_brevi; A_latis; L_canes; V_cymos; H_mutab; M_latif*
**2**	2.0	---	389.1076	C_16_H_22_O_11_	389: 227 (C_10_H_11_O_6_); 209 (C_10_H_9_O_5_); 183 (C_9_H_11_O_4_)	Iridoid	Hexosyl iridoid derivative	*D_kuntz*
**3**	3.0	---	389.1080	C_16_H_22_O_11_	389: 227 (C_10_H_11_O_6_); 209 (C_10_H_9_O_5_); 183 (C_9_H_11_O_4_)	Iridoid	Hexosyl iridoid derivative	*D_kuntz*
**4**	4.7	---	363.1297	C_15_H_23_O_10_	363:201(C_9_H_13_O_5_)	Iridoid	Hexosyl iridoid derivative	*A_hirta*
**5**	6.5	---	345.1191	C_15_H_22_O_9_	345: 207 (C_11_H_11_O_4_); 189 (C_11_H_9_O_3_); 183 (C_9_H_11_O_4_)	Iridoid	Hexosyl iridoid derivative	*A_hirta*
**6**	6.6	---	345.1190	C_15_H_22_O_9_	345: 207 (C_11_H_11_O_4_); 189 (C_11_H_9_O_3_); 183 (C_9_H_11_O_4_)	Iridoid	Hexosyl iridoid derivative	*A_hirta*
**7**	9.0	301/322	353.0891	C_16_H_18_O_9_	353: 191 (C_7_H_11_O_6_)	Phenylpropanoid	chlorogenic acid	*I_chili; A_latis; S_hispi; D_kuntz; C_punct; V_cymos; M_latif*
**8**	10.4	---	413.1085	C_18_H_22_O_11_	413: 371 (C_16_H_19_O_10_); 251 (C_12_H_11_O_6_); 191 (C_10_H_7_O_4_)	Iridoid	Asperuloside	*D_kuntz*
**9**	11.5	---	405.1401	C_17_H_26_O_11_	405: 387 (C_17_H_23_O_10_); 243 (C_11_H_15_O_6_); 225 (C_11_H_13_O_5_)	Iridoid	Sanshiside methyl ester	*L_canes*
**10**	11.5	268	633.0719	C_27_H_22_O_18_	633: 463 (C_20_H_15_O_13_); 301 (C_14_H_5_O_8_); 275 (C_13_H_7_O_7_)	Hydrolized tannin	Corilagin	*S_hispi*
**11**	12.6	268	951.0787	C_41_H_28_O_27_	951: 933(C_41_H_25_O_26_); 765 (C_34_H_21_O_21_); 463 (C_20_H_15_O_13_); 301 (C_14_H_5_O_8_); 275 (C_13_H_7_O_7_)	Hydrolized tannin	Geraniin	*S_hispi*
**12**	12.8	301/327	515.1180	C_25_H_24_O_12_	515: 191 (C_7_H_11_O_6_); 179 (C_9_H_7_O_4_)	Phenylpropanoid	1,3 Dicaffeoylquinic	*I_chili; A_latis*
**13**	16.0	281/344	463.0859	C_21_H_20_O_12_	463: 301 (C_15_H_9_O_7_)	Flavonoid	Isoquercitrin	*A_hirta*
**14**	16.4	282	463.0900	C_21_H_20_O_12_	---	Flavonoid	Flavanone derivative	*A_hirta; C_punct*
**Peak**	Ret (min)	UV	[M-H]-	Molecular formula	MS/MS	Class	Compounds	*Plants*
**15**	17.4	270/345	739.2096	C_33_H_39_O_19_	---	Flavonoid	Flavonol-hexosyl-dideoxyhexosyl	*T_form; V_cymos*
**16**	17.9	270/344	477.1059	C_22_H_22_O_12_	477: 314 (C_16_H_10_O_7_); 299 (C_15_H_7_O_7_)	Flavonoid	Isorhamnetin-O-glucoside	*A_hirta*
**17**	18.0	270/346	461.0740	C_21_H_18_O_12_	461: 285 (C_15_H_9_O_6_)	Flavonoid	Kaempferol-O-glucuronide	*L_canes; A_hirta; C_punct; V_cymos*
**18**	19.1	296/327	623.1958	C_29_H_36_O_15_	623: 461 (C_20_H_29_O_12_); 315 (C_14_H_19_O_8_); 179 (C_9_H_7_O_4_); 161 (C_9_H_5_O_3_)	Phenylpropanoid	Verbascosideo	*L_canes; H_serra*
**19**	19.1	301/327	515.1190	C_25_H_24_O_12_	515: 191 (C_7_H_11_O_6_); 179 (C_9_H_7_O_4_); 173 (C_7_H_9_O_5_)	Phenylpropanoid	3,4 Dicaffeoylquinic	*I_chili; A_latis; C_punct; V_cymos*
**20**	19.8	301/327	515.1193	C_25_H_24_O_12_	515: 191 (C_7_H_11_O_6_); 179 (C_9_H_7_O_4_)	Phenylpropanoid	3,5 Dicaffeoylquinic	*I_chili; A_latis; C_punct; V_cymos*
**21**	19.9	283/343	593.1489	C_27_H_30_O_15_	593: 285 (C_15_H_9_O_6_)	Flavonoid	Kaempferol-O-rutinoside	*I_chili; S_hispi; D_kuntz; H_mutab; H_brevi; C_acule*
**22**	20.0	270	419.0970	C_20_H_20_O_10_	419: 179 (C_9_H_7_O_4_)	Unknown	Unknown	*H_serra*
**23**	20.3	290/327	623.1973	C_29_H_36_O_15_	623: 461 (C_20_H_29_O_12_); 315 (C_14_H_19_O_8_); 179 (C_9_H_7_O_4_); 161 (C_9_H_5_O_3_)	Phenylpropanoid	Isoverbascosideo	*L_canes*
**24**	20.5	266/338	445.0753	C_21_H_18_O_11_	445: 269 (C_15_H_9_O_5_)	Flavonoid	Apigenin-O-glucuronide	*L_canes*
**Peak**	Ret (min)	UV	[M-H]-	Molecular formula	MS/MS	Class	Compounds	*Plants*
**25**	20.6	292/326	359.0760	C_18_H_16_O_8_	359: 197 (C_9_H_9_O_5_); 179 (C_9_H_7_O_4_); 161 (C_9_H_5_O_3_)	Phenylpropanoid	Rosmarinic acid	*H_mutab; H_brevi*
**26**	23.5	300/326	307.0462	C_14_H_12_O_8_	---	Phenylpropanoid	Unknown	*E_panic*
**27**	23.6	287/330	717.1420	C_36_H_30_O_16_	717: 519 (C_27_H_19_O_11_); 339 (C_18_H_11_O_7_); 321 (C_18_H_9_O_6_)	Unknown	Unknown	*H_mutab; H_brevi*
**28**	28.2	---	809.4308	C_42_H_66_O_15_	809: 603 (C_35_H_55_O_8_)	Triterpene saponin	quinovic acid derivative	*T_formo*
**29**	28.7	300/326	403.1380	C_21_H_24_O_8_	403: 359 (C_20_H_23_O_6_); 241 (C_12_H_17_O_5_); 197 (C_11_H_17_O_3_); 179 (C_9_H_7_O_4_)	Brevipolide	Dihydro-Brevipolide C	*H_brevi*
**30**	29.5	281/346	359.0759	C_18_H_16_O_8_	359: 329 (C_16_H_9_O_8_); 301 (C_15_H_9_O_7_); 286 (C_14_H_8_O_7_);	Flavonoid	5,6,3'-trihydroxy-3,7,4'-trimethoxyflavone	*H_brevi*
**31**	30.4	---	955.4908	C_48_H_76_O_19_	---	Triterpene saponin	Calenduloside derivative	*A_latis*
**32**	30.6	---	793.4358	C_35_H_55_O_7_	793:587 ()	Triterpene saponin	---	*T_formo; V_cymos*
**33**	30.8	300/313	387.1445	C_21_H_24_O_7_	403: 343 (C_20_H_23_O_5_); 241 (C_12_H_17_O_5_); 197 (C_11_H_17_O_3_); 179 (C_9_H_7_O_4_)	Brevipolide	Dihydro-Brevipolide F	*H_brevi*
**34**	31.8	---	955.4911	C_48_H_76_O_19_	---	Triterpene saponin	Calenduloside H	*I_chili; A_latis*
**35**	32.6	---	939.4971	C_48_H_76_O_18_	---	Triterpene saponin	Triterpene saponin derivative	*I_chili; A_latis*
**36**	34.8	---	793.4362	C_42_H_66_O_14_	---	Triterpene saponin	Ladyginoside B	*I_chili*
**37**	36.0	---	777.4416	C_42_H_66_O_13_	---	Triterpene saponin	Triterpene saponin derivative	*I_chili; A_latis*

**Fig 1:**
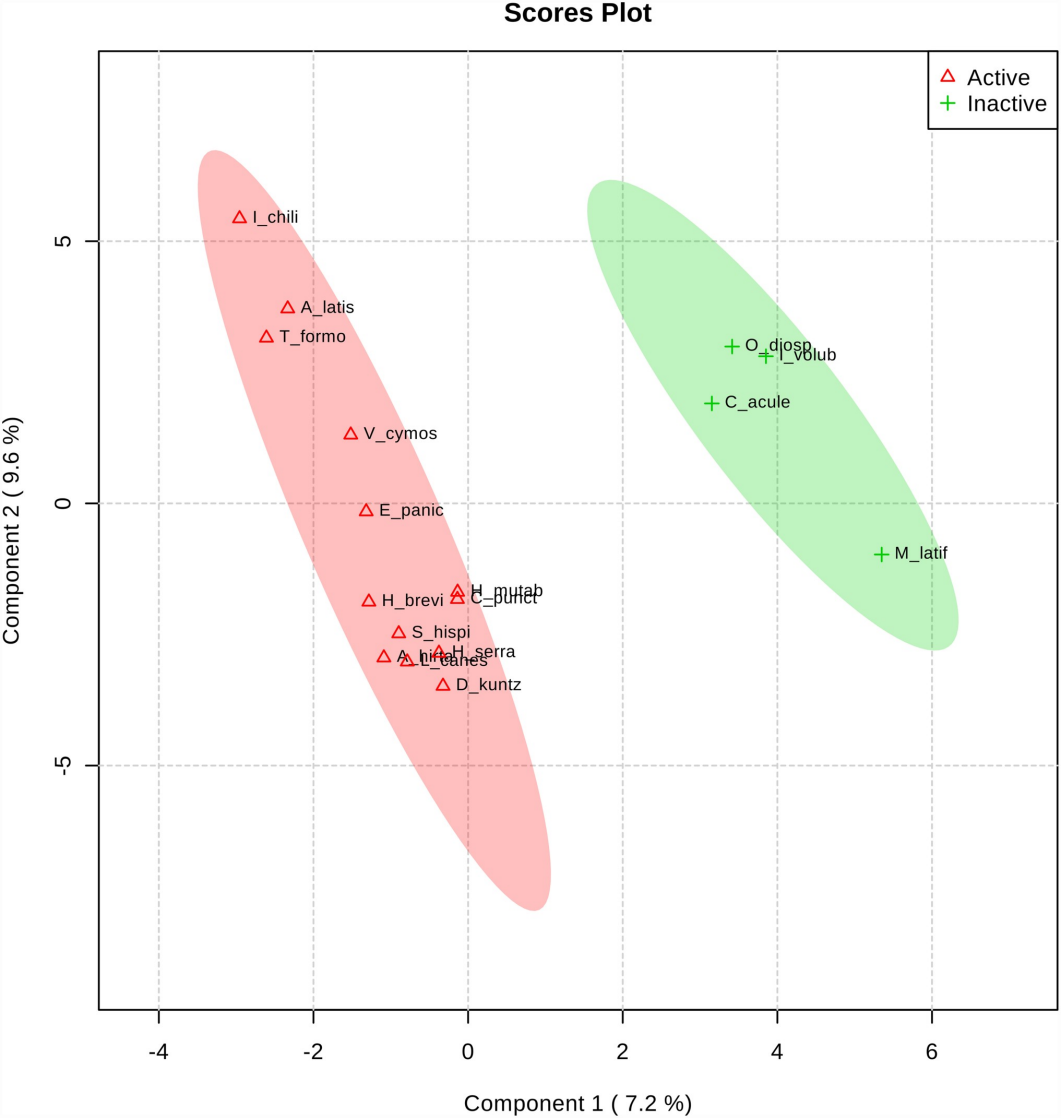
Geometric profile of active (triangle) and inactive (plus sign) fraction. Dispersion graph of PLS-DA points (each point represents ethanol extract of plant species).

The PLS-DA separated active and inactive species (Fig 1). It is noteworthy that some classes of metabolites found in active plants were similar and not found in the inactive plants. The PLS-DA was a robust method, with values above 0.7 for both R^2^ and Q^2^, which proves that the results were reliable.

**Fig 2:**
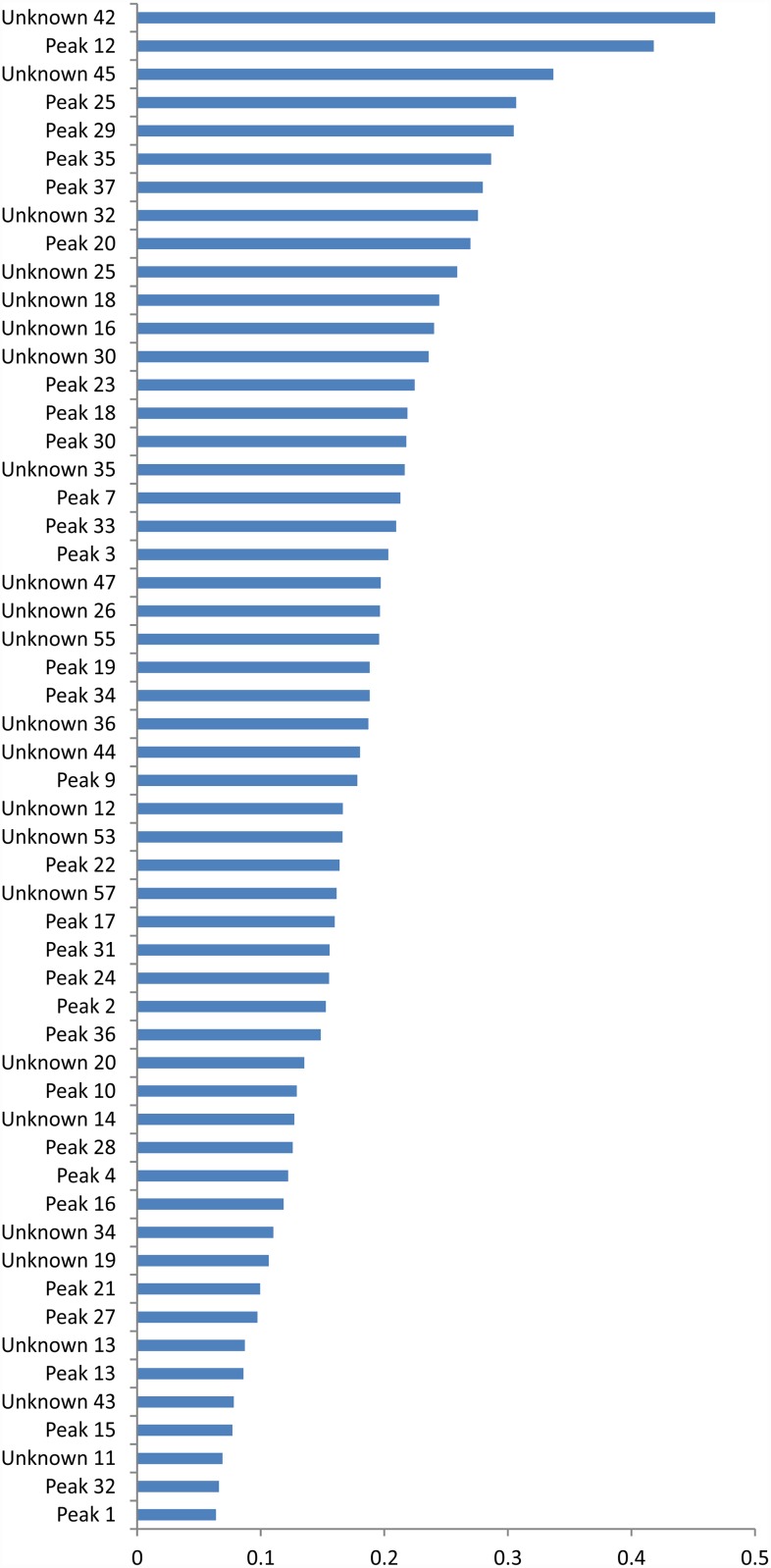
Correlation between secondary metabolites represented by peak observed in mass spectrometry and biological activity of extracts of origin characterized based on EC_90_.

The correlation analysis between compounds and EC_90_ revealed compounds that were positively correlated to ovicidal activity. Among the ten metabolites with better correlation coefficients, six were phenylpropanoids, two were triterpene saponins, one was a brevipolide and one was a flavonoid (Fig 2). Peak 12 and 20 (exhibiting an intense ion at *m/z* 515 [M-H] ^−^ compatible with C_25_H_24_O_12_), peak 25 (*m/z* 359.0760 [M-H]^−^ compatible with C_18_H_16_O_8_), peaks 18 and 23 (*m/z* 623 [M-H]^−^ compatible with C_29_H_36_O_15_) and peak 7 (*m/z* 353.0891 [M-H]^−^ compatible with C_16_H_18_O_9_) were the phenylpropanoids with the best correlation to biology activity (EC_90_) and were putatively identified as 1,3 dicaffeoylquinic, 3,5 dicaffeoylquinic, rosmarinic acid, verbascoside, isoverbascoside and chlorogenic acid, respectively. Peak 29 exhibited intense ions at *m/z* 403.1380 [M-H]^−^ compatible with C_21_H_24_O_8_ and was putatively identified as dihydro-brevipolide C. Peak 4 (*m/z* 939.4971 [M-H]^−^ compatible with C_48_H_76_O_18_) and 5 (intense ions at *m/z* 777.4416 [M-H]^−^ compatible with C_42_H_66_O_13_) were putatively identified as triterpene saponin derivatives. Peak 9 (*m/z* 359. 0759 [M-H]^−^ compatible with C_18_H_16_O_8_) was putatively identified as 5, 6, 3’-trihydroxy-3,7,4’trimethoxyflavone. All compounds listed as unknown in Fig 2 showed a very low intensity (lower than 20000) in the mass spectrometry analysis and was not possible to identify these compounds. They listed the information of this lower intensity compounds separately as supplementary material (S1 Table) to not overload Table 4. Also, chromatograms and MS/MS spectral data that allowed the identification of studied metabolites are presented as supplementary data (S1 and S2 Figs, respectively).

**Fig 3:**
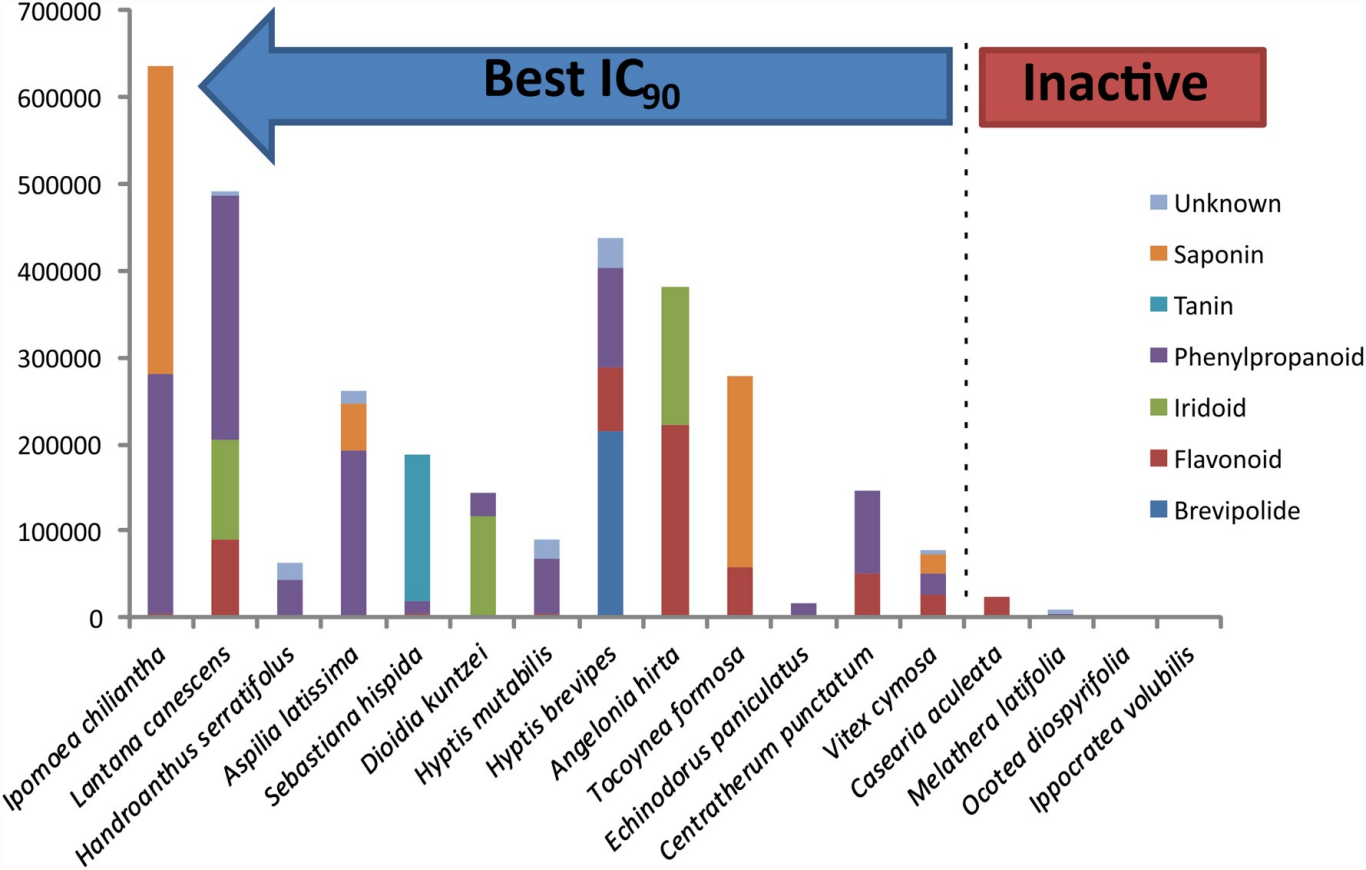
Chemical constitution (classes of major compounds) of ethanol extracts from 17 plant species evaluated for ovicidal action against *H*. *placei*. Plant species ordered based on ovidical activity characterized by EC_90_ (calculated for active plants plotted in *I_chili*–*V_cymos* range) from right (less active) to left (more active).

The plant with the greatest ovicidal activity (*I*. *chiliantha*) was composed of phenylpropanoids (chlorogenic acid, 1,3 dicaffeoylquinic, 3,4 dicaffeoylquinic and 3,5 dicaffeoylquinic), triterpene saponins (calenduloside H, Triterpene saponin derivative and ladygenoside B) and a flavonoid (kaempferol-O-rutinoside) as the main metabolites (Fig 3). Other combinations of metabolites that had considerable ovidal activity were flavonoids (kaempferol-O-glucuronide and apigenin-O-glucuronide), iridoid (sanshiside methyl ester) and phenylpropanoids (verbascoside and isoverbascoside) combined in the same extract (*L*. *canescens*) and saponins (calenduloside derivative, calenduloside H and Triterpene saponin derivative) combined with phenylpropanoids (chlorogenic acid, 1,3 dicaffeoylquinic, 3,4 dicaffeoylquinic and 3,5 dicaffeoylquinic) in the extract from *A*. *latissima*. Ethanolic extracts of the species *I*. *chiliantha*, *L*. *canescens* and *A*. *latissima* were among the most active (lowest EC_90_).

## Discussion

Despite the complex chemical profile of the species studied, it was possible to align the data with the aid of a pool of samples. The choice of univariate correlation methods is justified by the fact that we can directly list the main determinant compounds for biological activity and thus direct future research or studies aimed at the production of extracts enriched with these substances. A recent study has demonstrated the efficacy of this technique for the determination of active compounds from propolis extracts on biofilms of *Staphylococcus aureus* and *Trichomonas vaginalis* [24]. In the present work, the positive correlation of the same classes of compounds in plants belonging to different genera and families reinforces the robustness of the statistical data and demonstrates the efficacy of the proposed tool for the selection of bioactive compounds without the need for the isolation and reevaluation. This method also enables determining synergism among classes of compounds, which would be impracticable using traditional methods.

Some of the metabolites identified in the present study have been associated with nematicidal action. Flavonoids (flavones, flavanones, and flavonols) and their aglycone and glycosidic derivatives have been described as having nematicidal action against different species and stages of the lifecycle of ruminant GINs [25,26,27]. Ovicidal activity against *Haemonchus contortus* has been described for flavonoids from *Spiglia anthelmia* [12] and the flavonol derivatives quercetin and flavone apigenin extracted from *Artemisia campestres* (reviewed by [27]). A homoisoflavonoid from *Agave sisalana* significantly reduced the larval hatchability of nematode parasites of goats (*Haemonchus* spp., *Oesophagostomum* and *Trichostrongylus* spp.) [25]. Moreover, flavonoids have been also associated with the inhibition of larval development [28], inhibition of larval exsheathment (naringenin aglycone, kaempferol, myricetin, luteolin and quercetin) ([29], reviewed by [27]), inhibition of larval migration [30] and a reduction in the motility of *H*. *contortus* adults [27].

While the anthelmintic action attributed to the class of flavonoids has been previously reported, not all flavonoids are associated with antiparasitic action, as there is no clear relationship between structure and anthelmintic activity [27]. Besides the molecular structure, other important factors that may influence the activity of flavonoids include the concentration in contact with the parasite and the concomitant presence of other secondary metabolites, which can act in a complementary manner through by different mechanisms of action in the improvement of the solubility (surfactant properties) or stability (antioxidant effect) of other compounds. Klongsiriwet et al. [26] reported the synergistic effect between tannins and flavonoids, as evidenced by an increase in the effect of procyanidins and prodelphinidins (condensed polyphenol derivatives) on the inhibition of larval exsheathment in *H*. *contortus* when associated with quercetin or luteolin [12].

Our results suggest that the presence of more than one class of compounds is necessary for high ovicidal activity. This can be seen in *C*. *aculeata*, which, despite accumulating flavonoid 21, was shown to be inactive, which may be related to the absence of other classes of compounds. Reinforcing this observation, the most active species (based on EC_90_) were those with more than one class of metabolites (*Ipomoea chiliantha*, *Lantana canescens*, and *Handroanthus serratifolius*), demonstrating an evident joint action.

The mechanism of action of flavonoids with regard to anthelmintic performance is not known. However, these compounds seem to act in a similar way to tannins, likely interacting with proteins from non-covalent hydrophobic bonds, which may result in disruption and inhibition. Unlike tannins, flavonoids can be easily internalized by helminths and can reach active sites that are inaccessible to tannins [27].

Tannins are polyphenolic compounds with known anthelmintic action and are classified according to their chemical structure as condensed tannins, which are the most widely studied metabolites and are mainly those contained in tannin-rich forages [31], and hydrolyzable tannins. In the present study, only hydrolyzable tannins were detected (ethanol extract from *S*. *hispida*). It is noteworthy that although the method used for the detection of secondary metabolites (HPLC-MS) is not the most suitable for detecting high molecular weight compounds, such as condensed tannins, we believe that this class was not present in any extract, since the precursors (catechin and isocatechin monomers) were not found.

The ability of tannins to bind to proteins is well known (reviewed by [11]) and this characteristic has been implicated in the anthelmintic action of these compounds. The ovicidal effect and reduction in the motility of adult parasites of the genus *Haemonchus* had been demonstrated for ellagic acid present in ellagitannins [32]. The ovicidal action of ellagitannins is associated with the ability of tannins to interact with and precipitate proteins. Particularly in eggs, a coating of tannin-protein complexes forms that is bound to the eggshell and prevents hatching [33]. In the present study, corilagin and geraniin were detected in the extract from *Sebastiana hispida* and were associated with the ovicidal action of the ethanol extract. The eggs treated with the plant extract exhibited an amorphous coating, with the presence of viable larvae at the end of the incubation period, but no hatching of these larvae was observed, which confirms with the previously described action for hydrolysable tannins in preventing larval hatchability [34]. Corilagin and geraniin have hexahydroxydiphenic acid in their chemical structure and release ellagic acid into the medium when undergoing hydrolysis [32].

Studies reporting the anthelmintic activity of saponins are scarce [34]. Saponins have been identified as part of the chemical composition of extracts that have nematicidal action against parasites of the genus *Haemonchus* [32,30]. Therefore, these compounds have been recognized for working together on tannins and flavonoids [30], facilitating the action of phenolic derivatives on proteins due to the increased permeability of cell membranes, which are destabilized by the action of saponins (reviewed by [34]). Specific studies confirming the nematicidal action of saponins were only conducted with the phytonematodes *Xiphinema index*, *Meloidogyne incognita* and *Globodera rostochiensis* [34,35]. In the present study, triterpene saponins from *Ipomoea chiliantha* (peaks 28, 34, 35, 36 and 37), *Tocoiena formosa* (peak 32) and *Aspilia latissima* (peaks 28, 31, 34, 35 and 37) were associated with ovicidal action. The extracts from *I*. *chiliantha* and *A*. *latissima* have saponins and phenylpropanoids (peaks 7, 12, 19 and 20) as the main metabolites, lending support to the notion that these two classes together are related to high ovicidal activity. *A*. *latissima*, which was one of the species with the highest activity, also accumulates phenylpropanoids and triterpene saponins.

The ethanol extract from *I*. *chiliantha* exhibited the same classes of metabolites as those found observed in *A*. *latissima*, but at higher concentrations and there was also an increase in flavonoid 21. This chemical difference may help explain the reduction in the EC50 from 1.28 mg/mL (95% CI 0.99–1.66) in *A*. *latissima* to 0.54 mg/mL (95% CI 0.35–0.82) in *I*. *chiliantha*. The data suggest increased ovicidal activity in the presence of higher concentrations of saponins (peaks 28, 34, 35, 36 and 37), phenylpropanoids (peaks 7, 12, 19 and 20) and flavonoid 21.

Few investigations have been carried out to determine the effect of phenylpropanoids on parasitic nematodes and data on nematicidal action are restricted to a small number of studies on fish, dog and swine parasites [27]. Recently, some phenylpropanoids have been evaluated with regard to the effects on nematodes of ruminant parasites; caffeic acid, p-coumaric acid, ferulic acid, methyl caffeate, methyl p-coumarate and methyl ferulate were identified as having ovicidal action [36] and chlorogenic acid was identified as having both ovicidal and larvicidal action [37] against *H*. *contortus*. The ovicidal effect of chlorogenic acid and its derivatives was also observed in the present study. This is the first report of the ovicidal effect of extracts containing these phenylpropanoids on *H*. *placei*. The concentration of monomeric and dimeric chlorogenic acid derivatives that enter into contact with the eggs of the parasite seems to be determinant for the improvement in activity, since this class was not able to inhibit larval hatchability satisfactorily at low concentrations, as verified for the ethanol extract from *Melanthera latifolia*, which was considered inactive in the present study. Other phenylpropanoids identified (rosmarinic acid, verbascoside and isoverbascoside) were also associated with ovicidal action for the first time. A similar condition was attributed to iridoids, which were evaluated for the first time on *H*. *placei*, and brevipolide, evaluated for the first time on nematodes. The evaluation of the anthelmintic potential of iridoids on ruminant GINs is restricted and only the iridoid aucubin has been cataloged for its transient inhibition of motility in third instar larvae of the bovine abomasum parasite *Ostertagia ostertagi* [38].

## Conclusion

The present investigation demonstrates that the metabolomic technique was efficient at detecting secondary metabolites with ovicidal activity against *H*. *placei*. Thus, the use of metabolomics can be a tool to accelerate and simplify bioprospecting research with plant extracts in veterinary parasitology. Moreover, it was demonstrated that phenylpropanoids were the compounds with a higher correlation to biological activity. Therefore, these metabolites are implicated in the ovicidal action against *H*. *placei*. Other classes, such as flavonoids and triterpene saponins, may act synergistically, increasing the activity of phenylpropanoids.

## Supporting information

S1 Fig Total ion chromatogram in the negative ion mode of extract of plants evaluated for ovicidal activity against *Haemonchus placei*. Number 1–37 were related to the compounds identified in the Table 4.
(PDF)

S2 Fig MS/MS spectra of the identified compounds (Table 4). (PDF)

S1 Table Retention time, mass, molecular formula, chemical class of compounds of Fig 2 that were also positively correlated to EC90 but with a relative intensity of masses lower than 20000. *[M-2H]-2.
(PDF)
