# Supplementary material for: Discovery of potential ovicidal natural products using metabolomics
Source: PLoS One. 2019 Jan 25;14(1):e0211237. doi: 10.1371/journal.pone.0211237 (PMC6347362; doi:10.1371/journal.pone.0211237)
Supplement: S1 Fig — Number 1–37 were related to the compounds identified in the Table 4. (PDF) [file pone.0211237.s001.pdf]

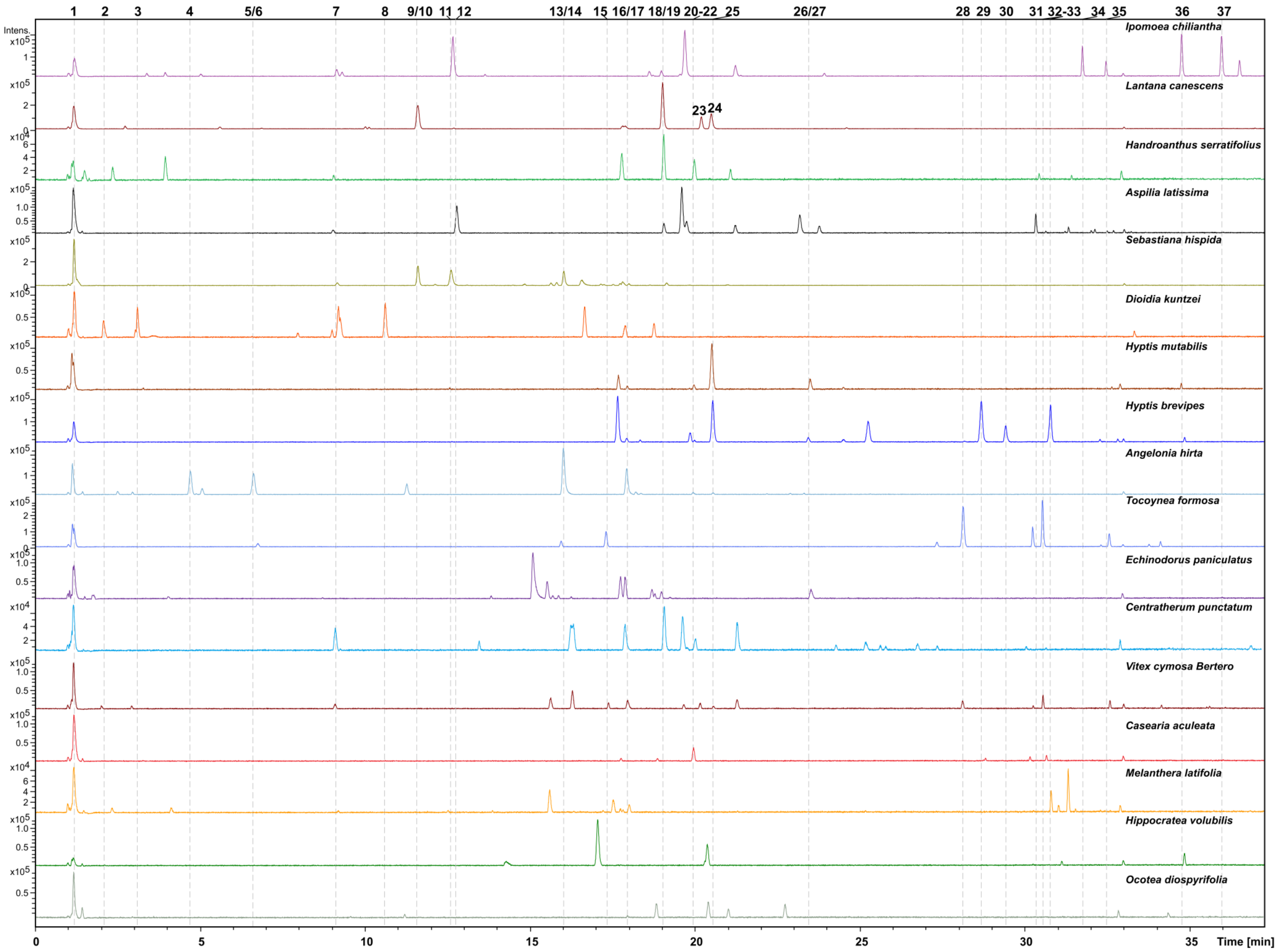

S1 Fig. - Total ion chromatogram in the negative ion mode of extract of plants evaluated for ovicidal activity against *Haemonchus placei*. Number 1-37 were related to the compounds identified in the table 4.
