# Supplementary figures and images for: Discovery of potential ovicidal natural products using metabolomics
Source: PLoS One. 2019 Jan 25;14(1):e0211237. doi: 10.1371/journal.pone.0211237 (PMC6347362; doi:10.1371/journal.pone.0211237)

**S2 Fig.** MS/MS spectra of the identified compounds (Table 4).

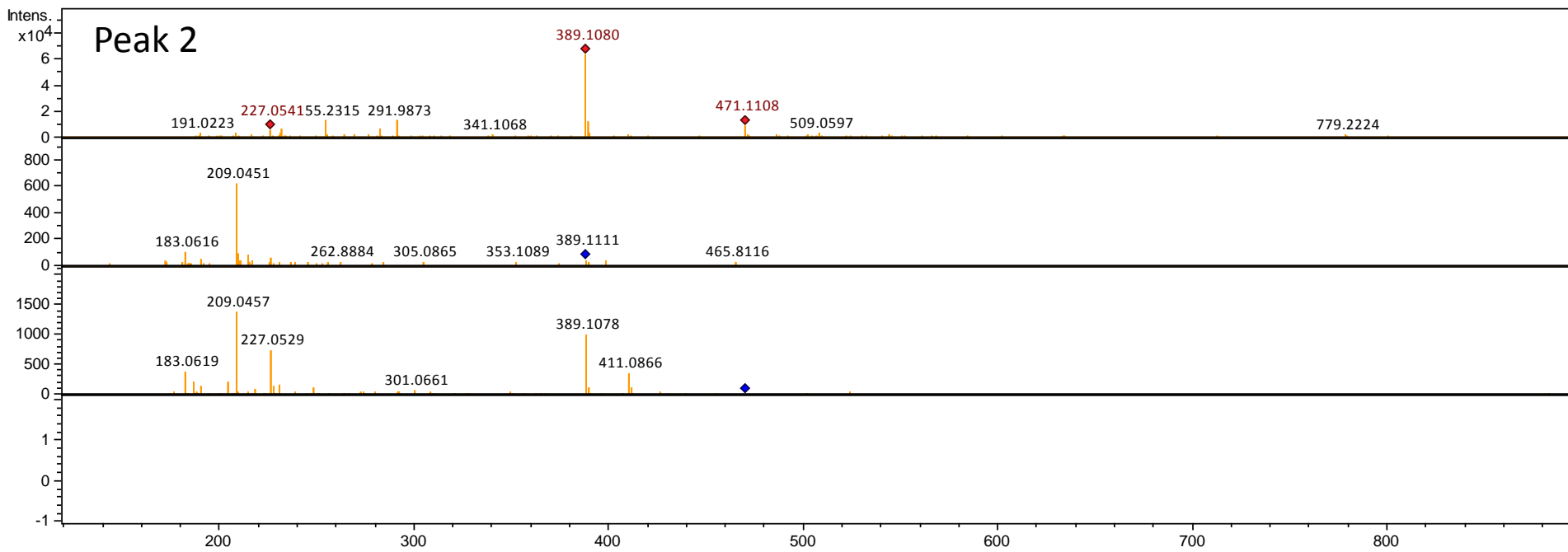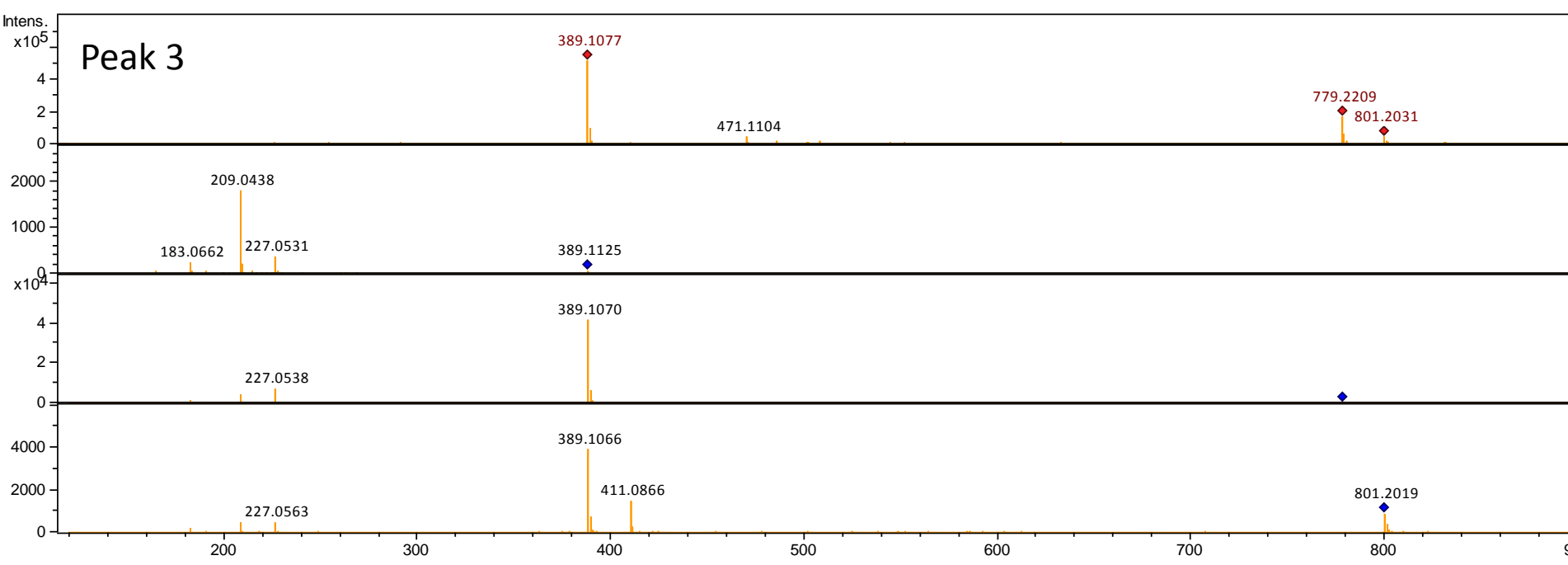

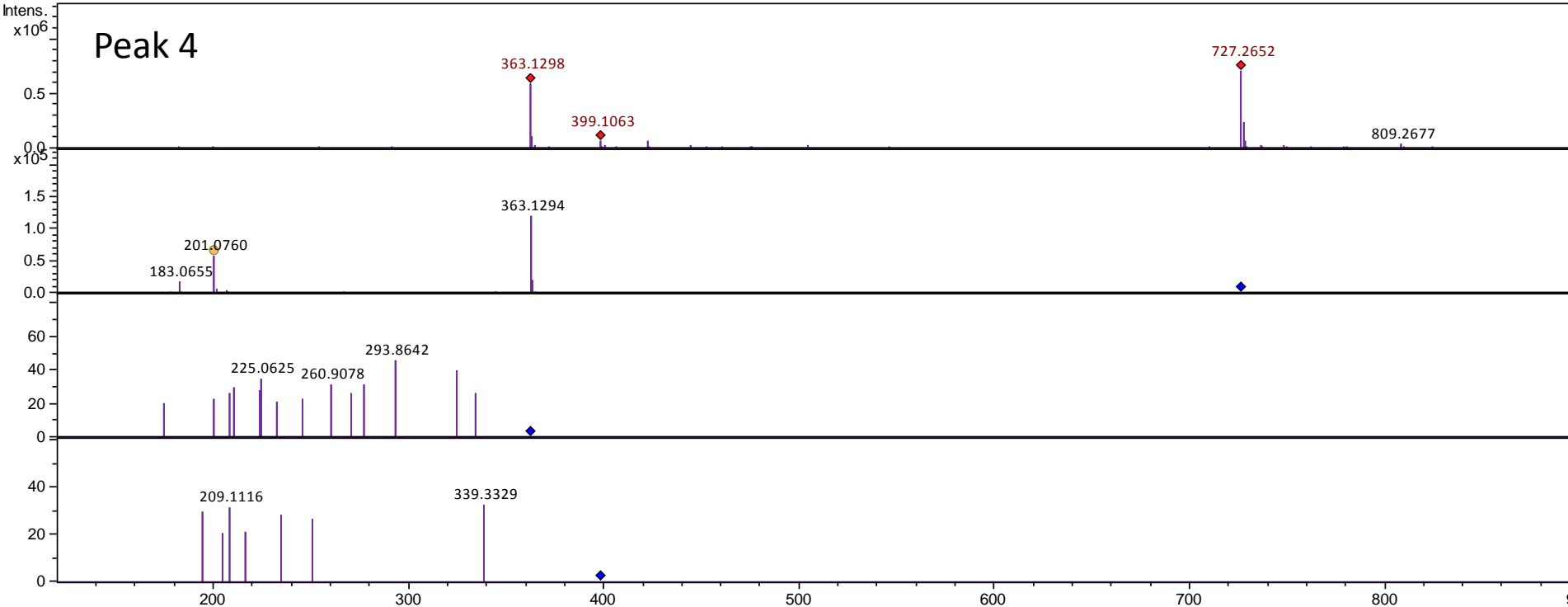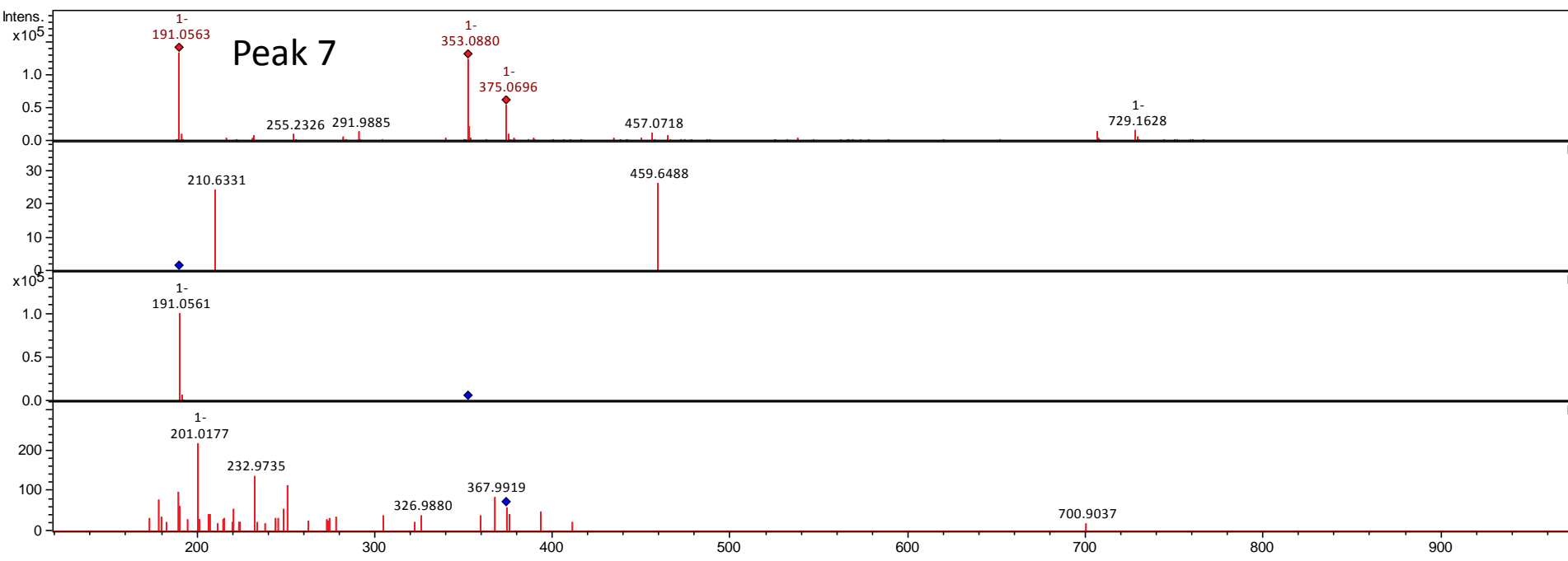

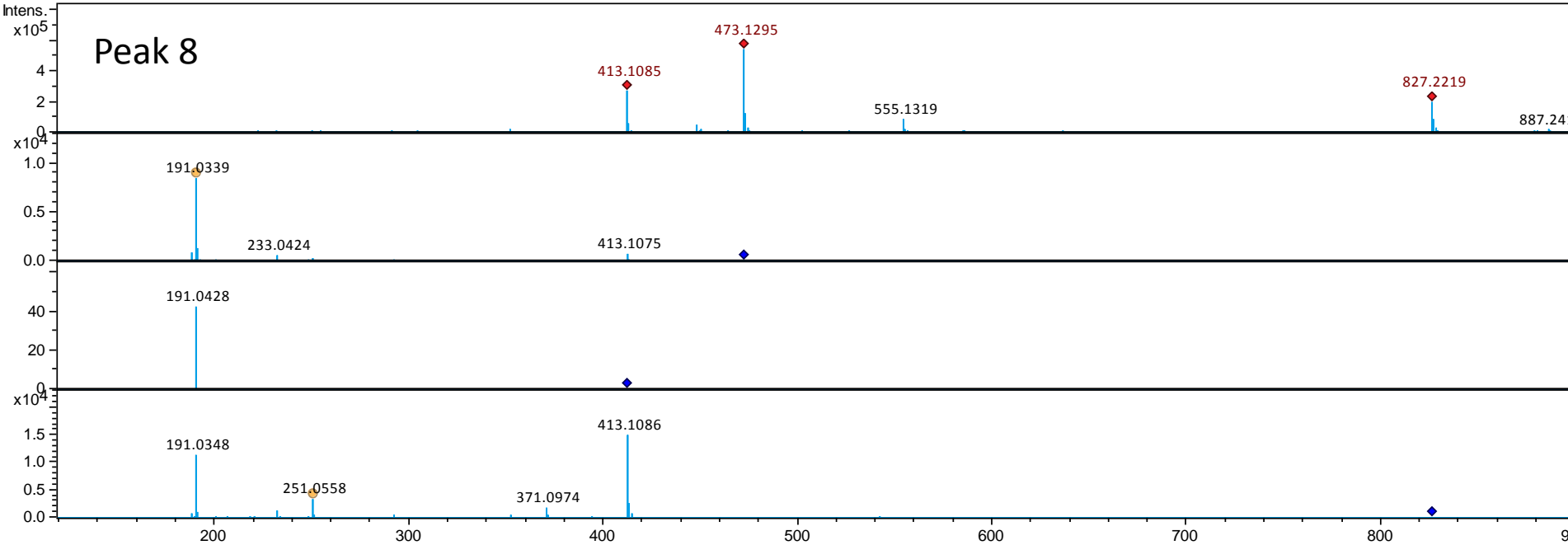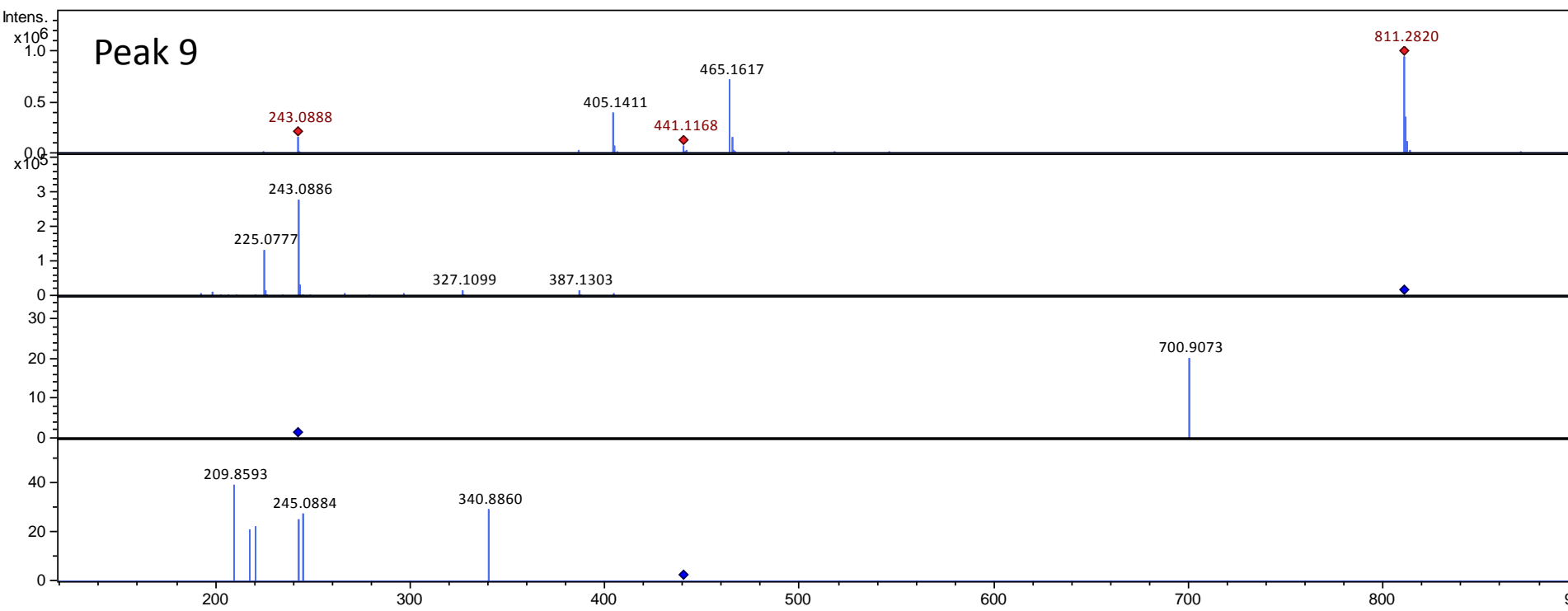

## Peak 10/11

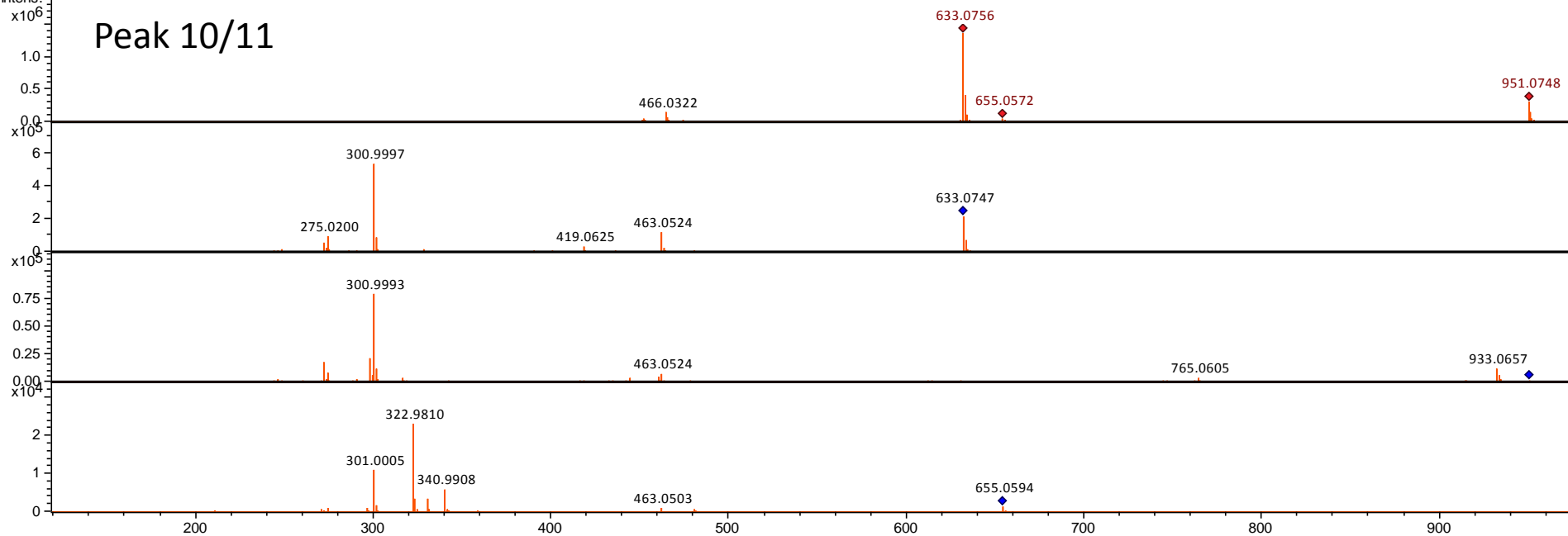

## Peak 12

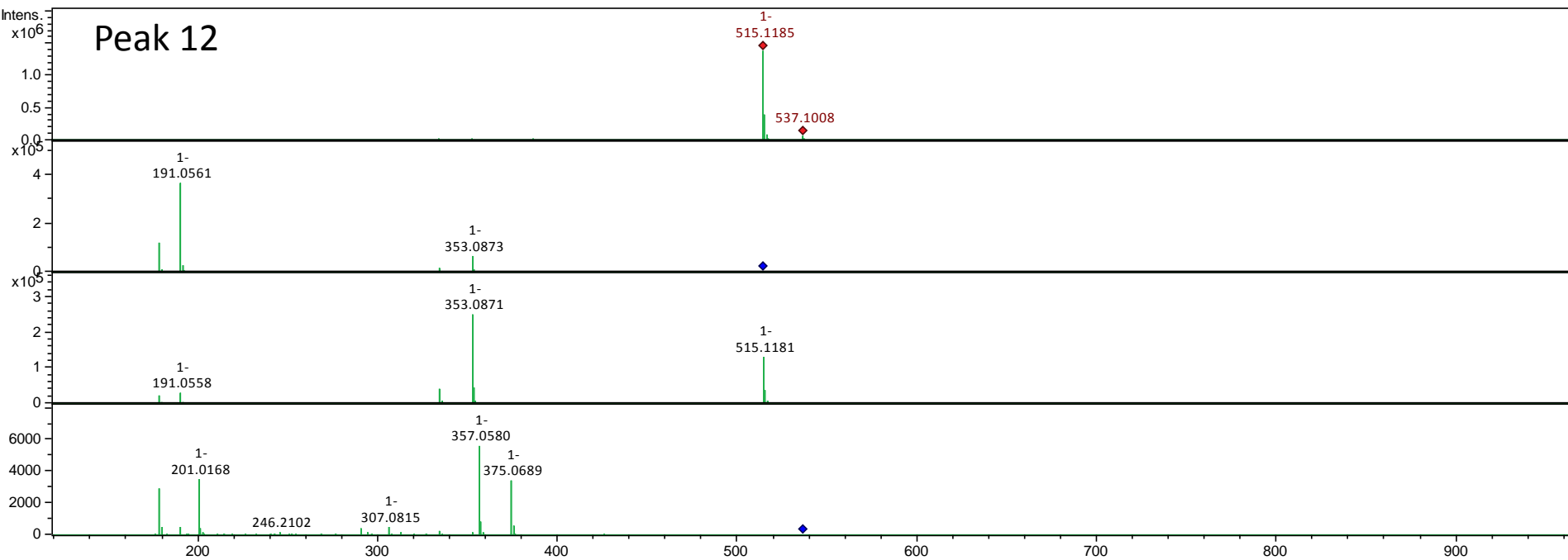

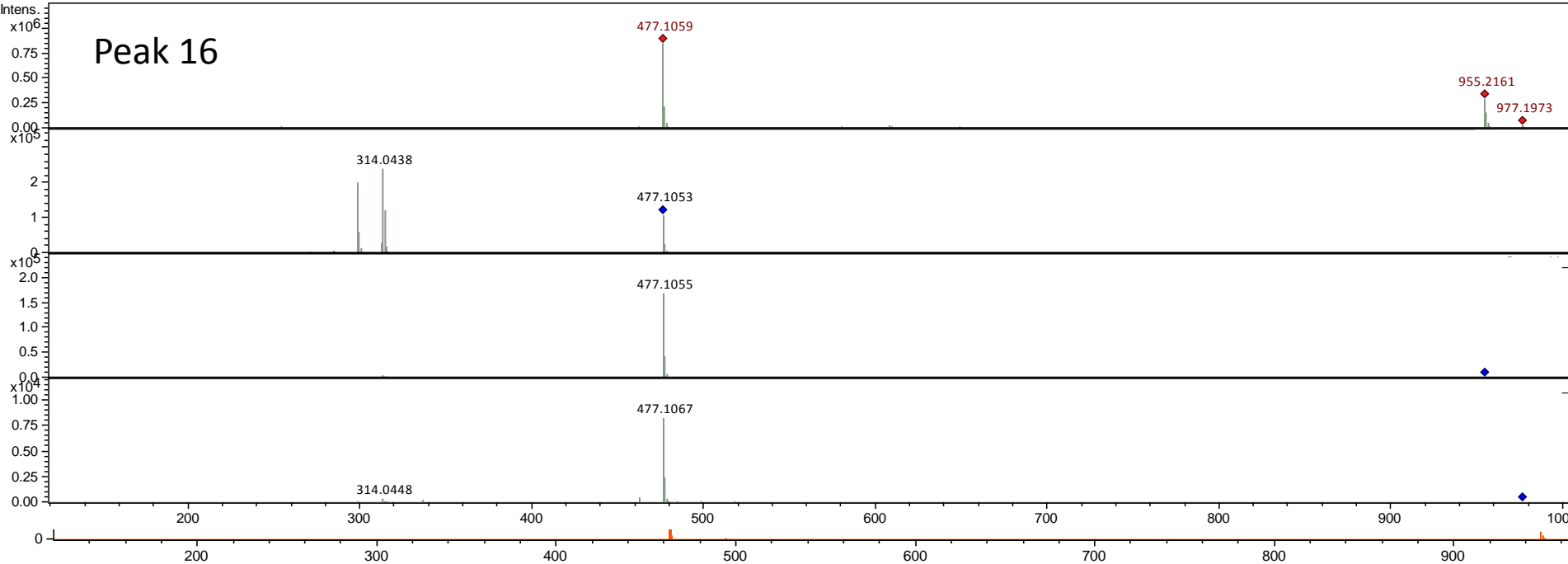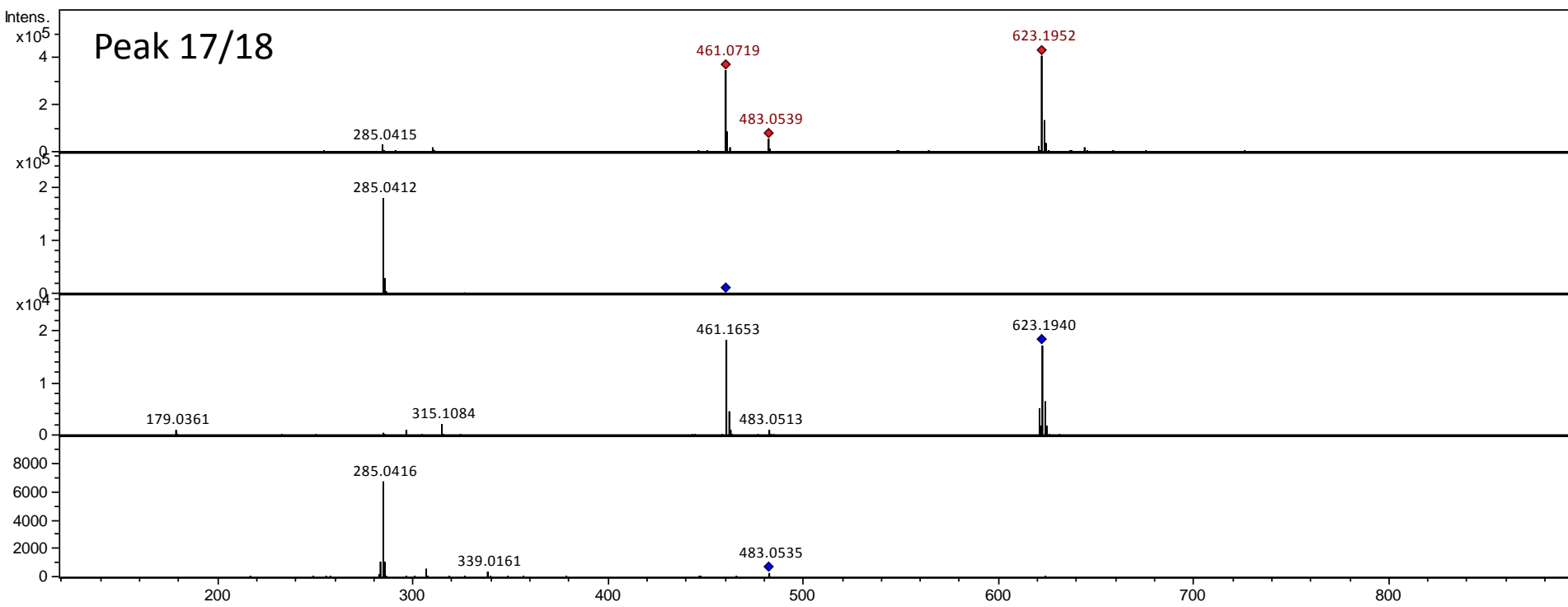

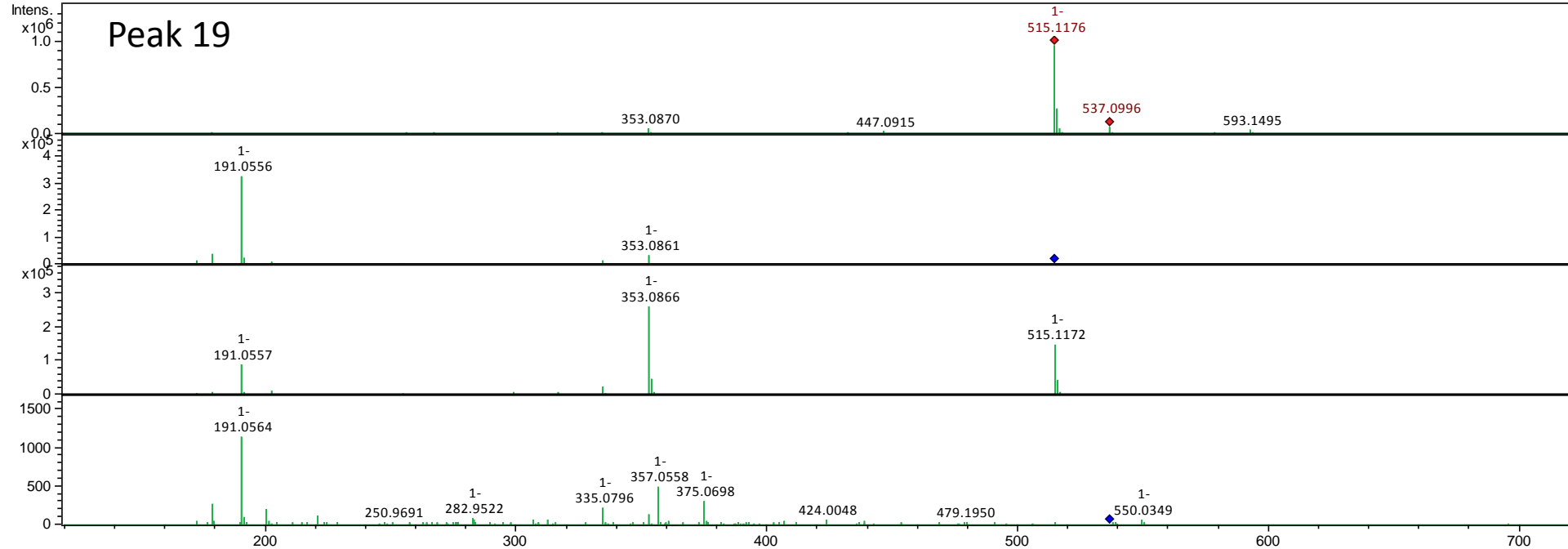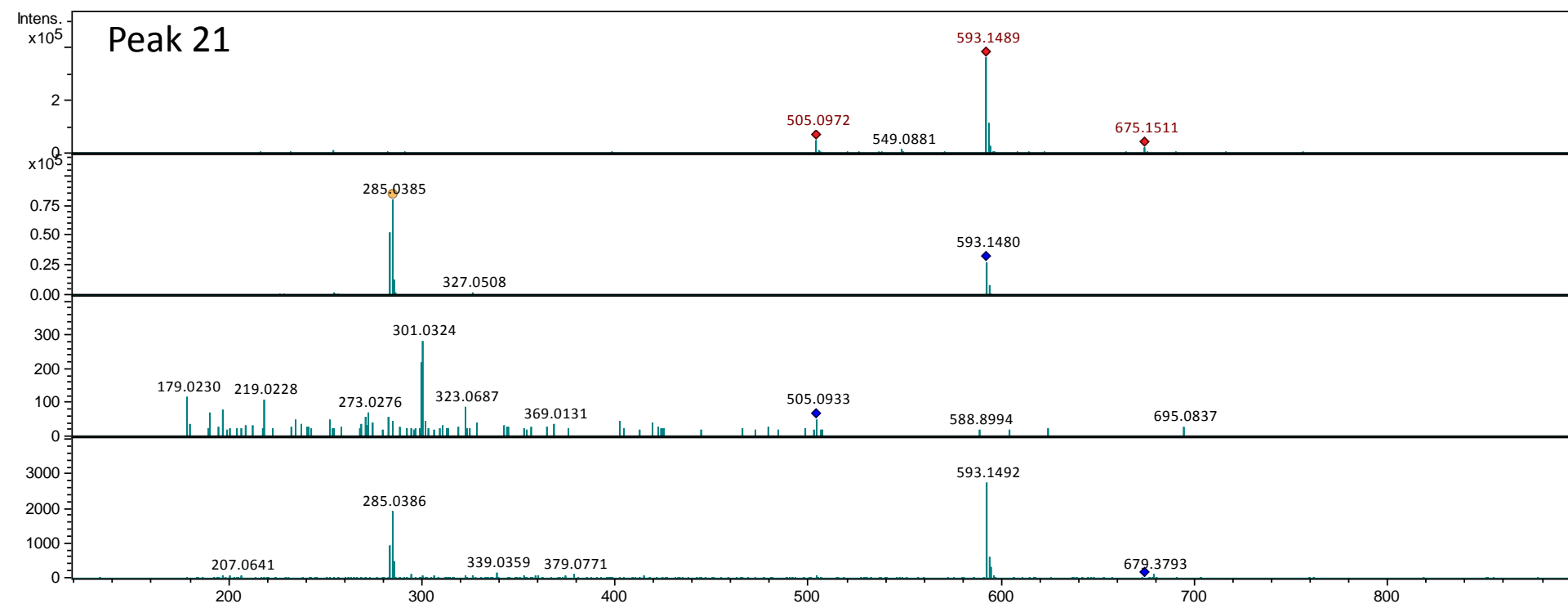

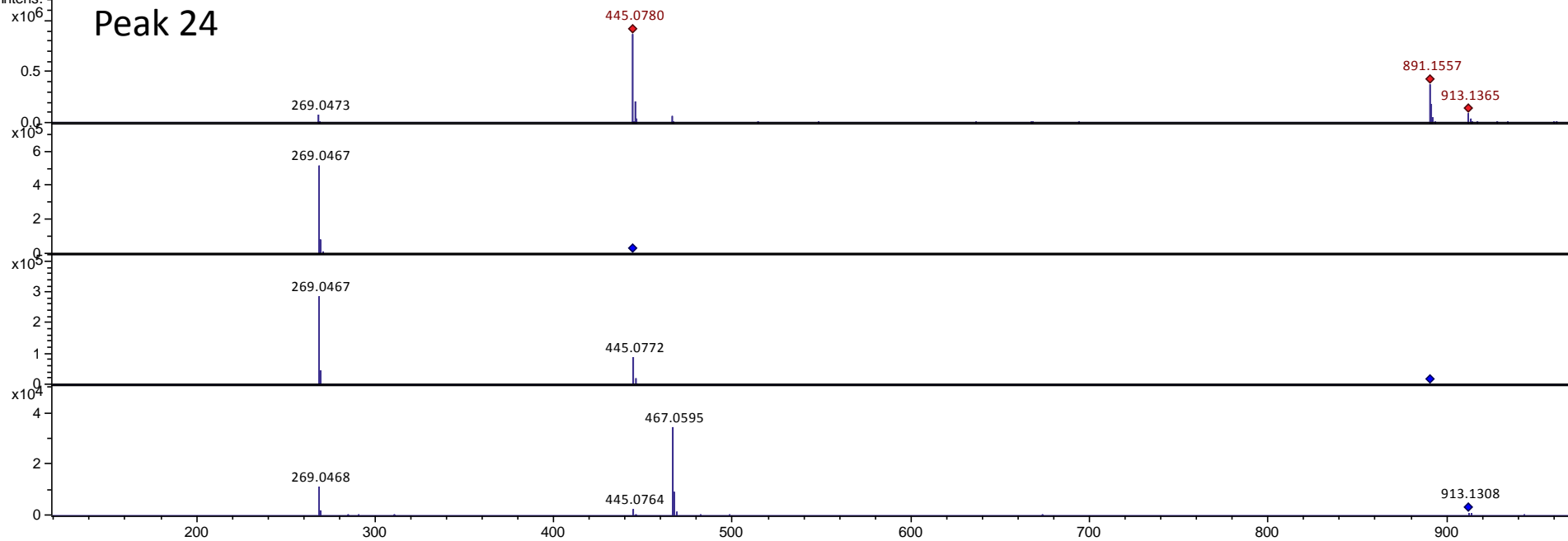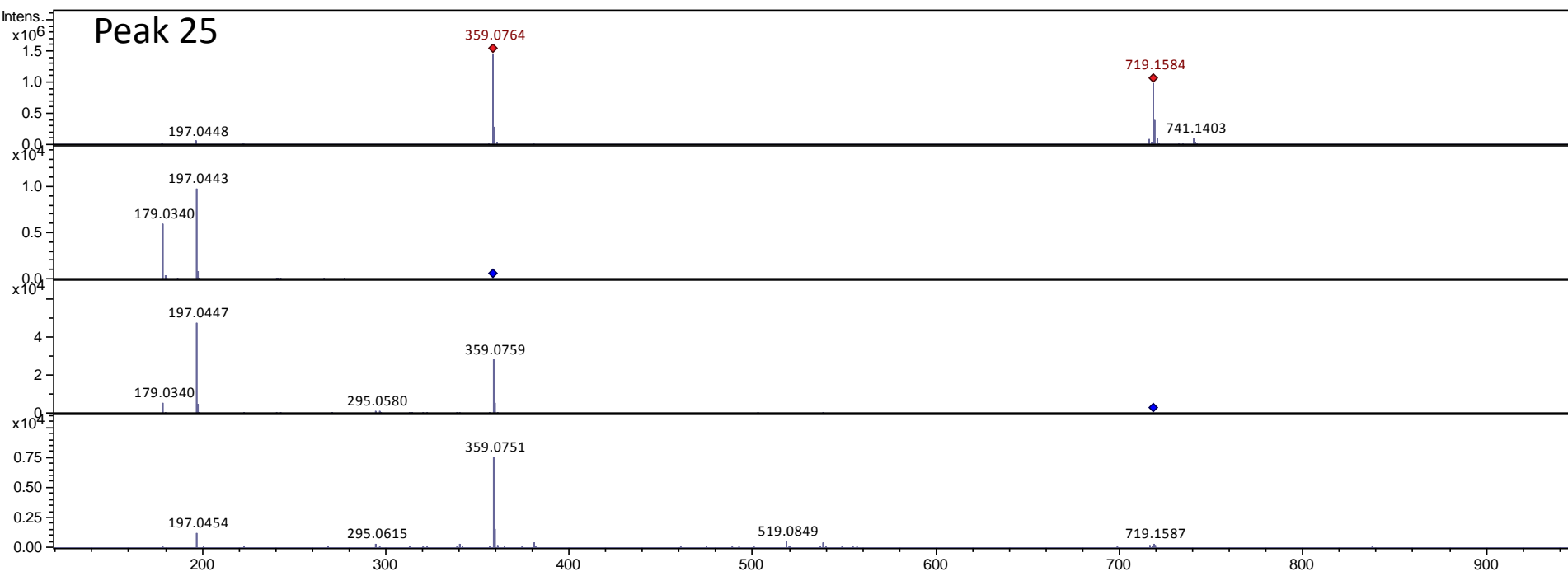

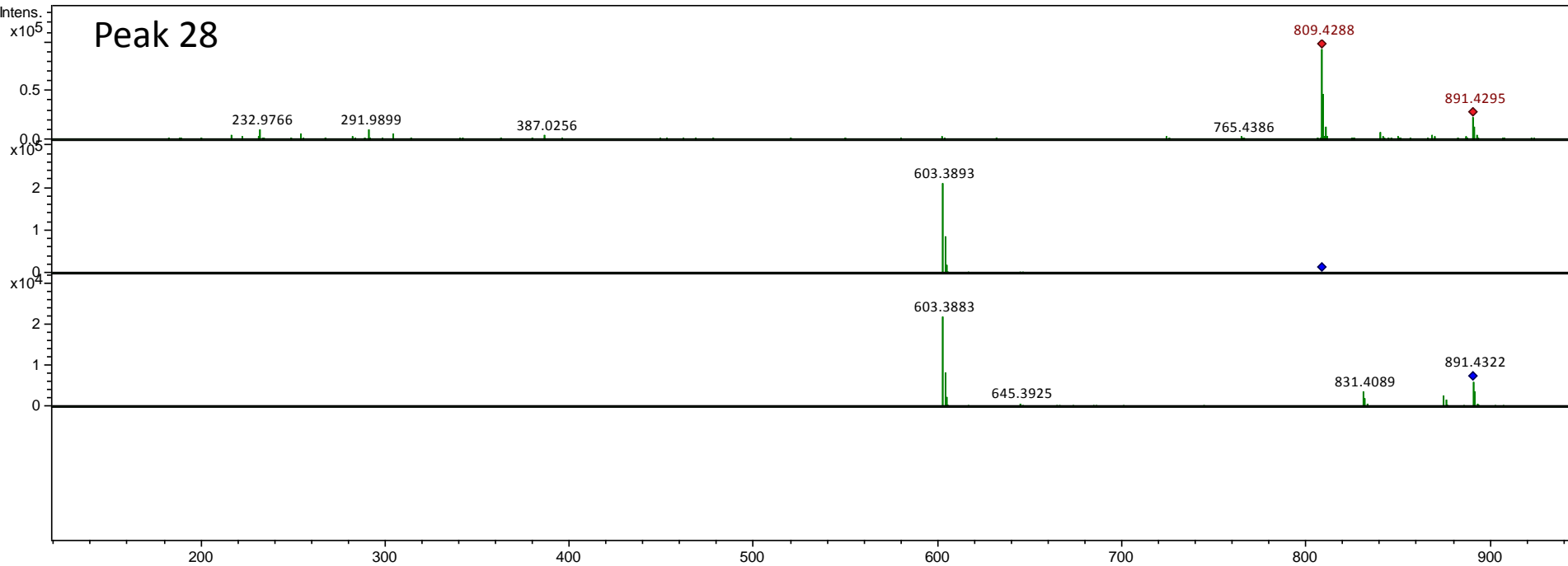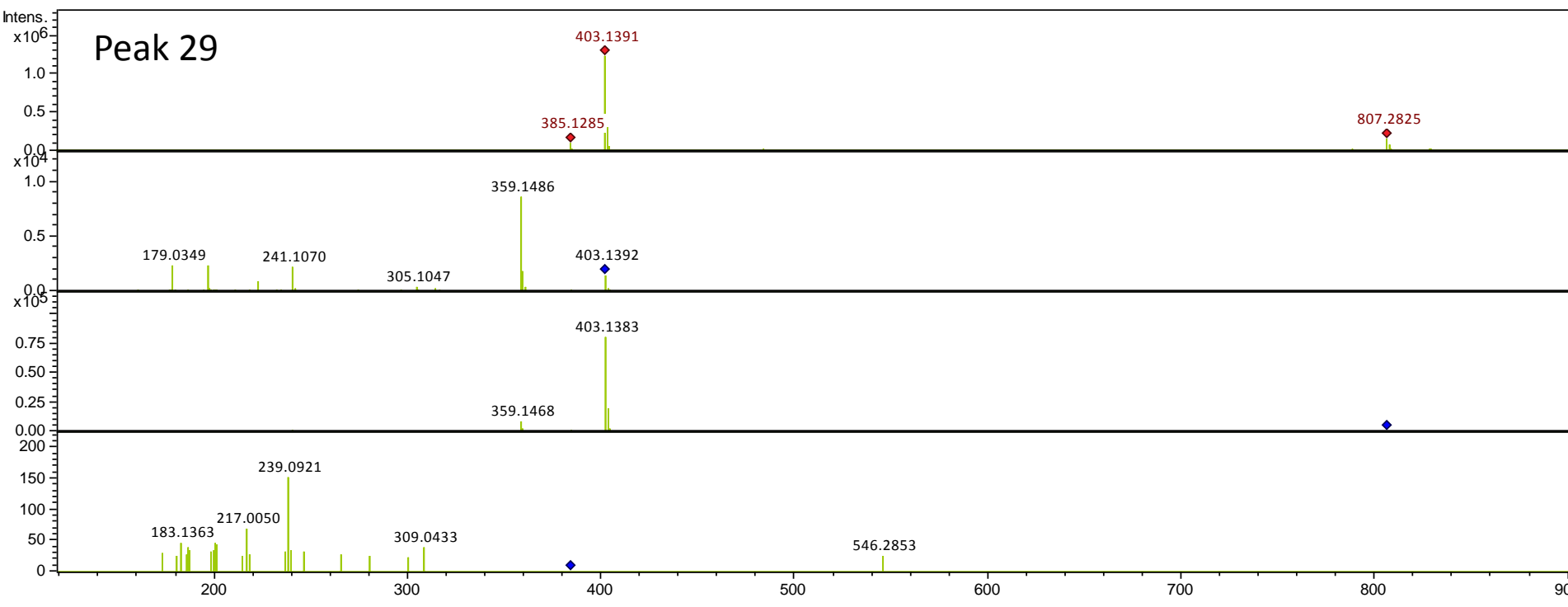

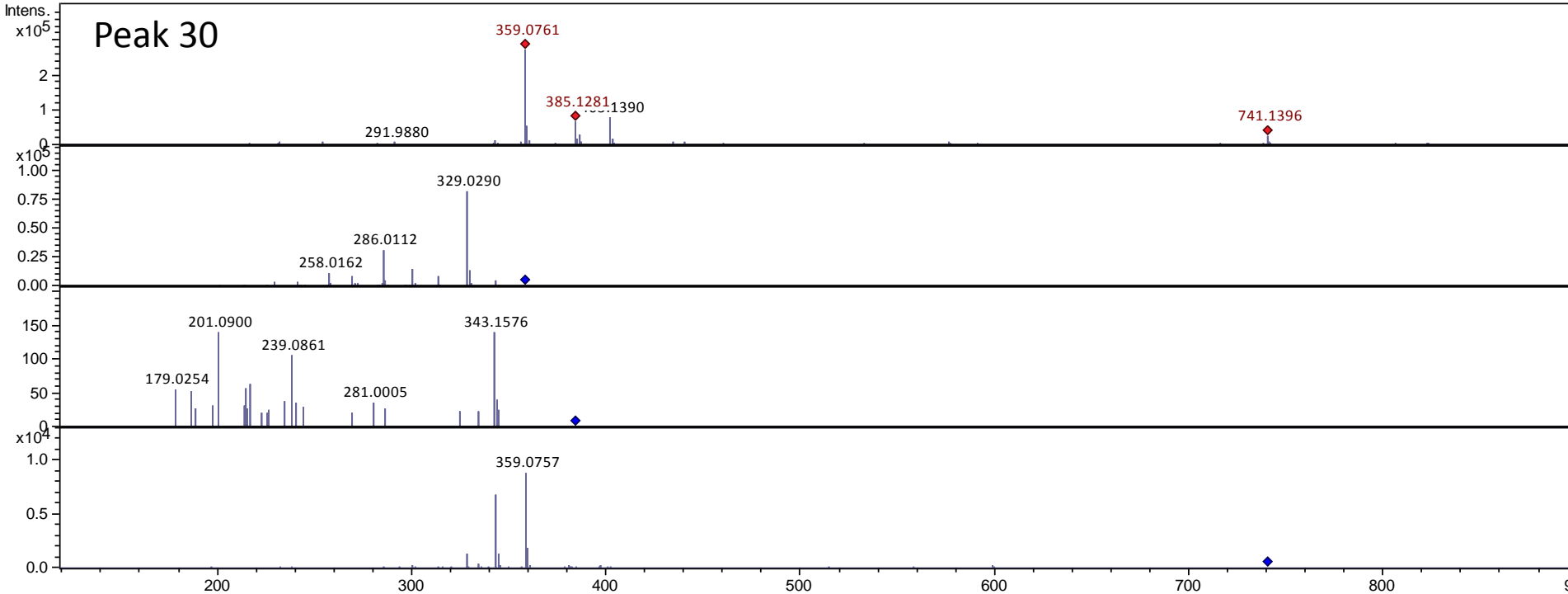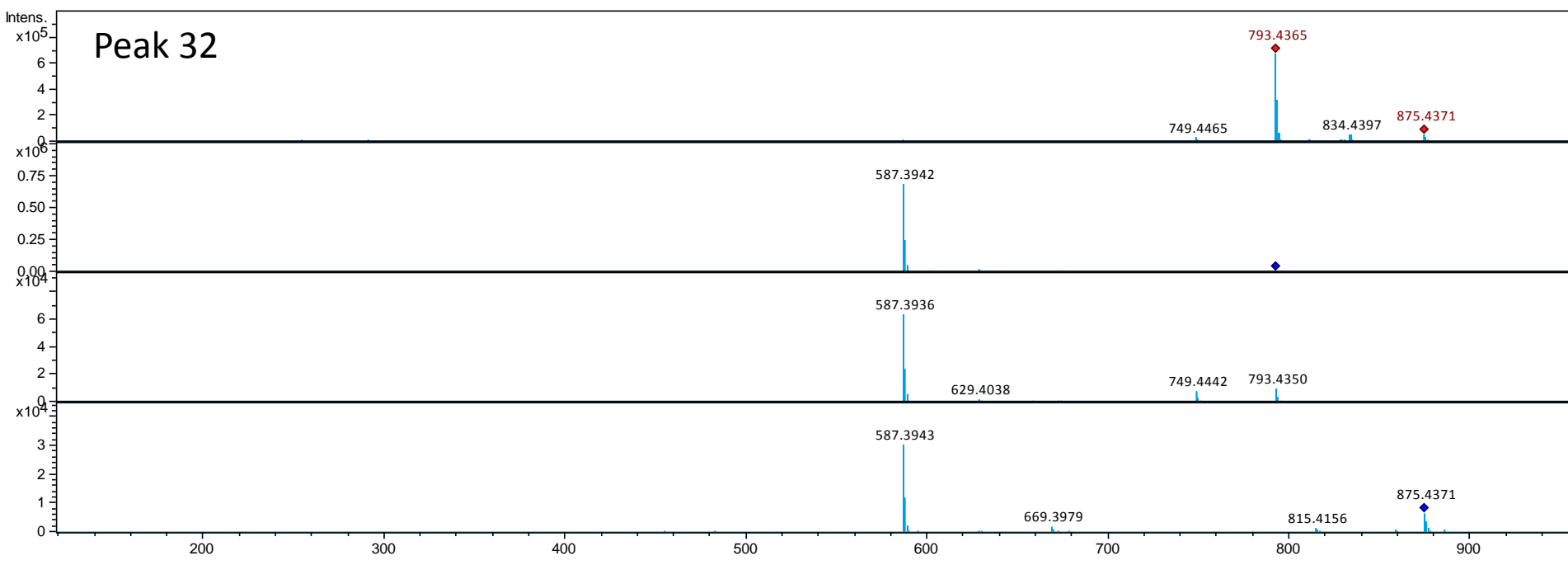

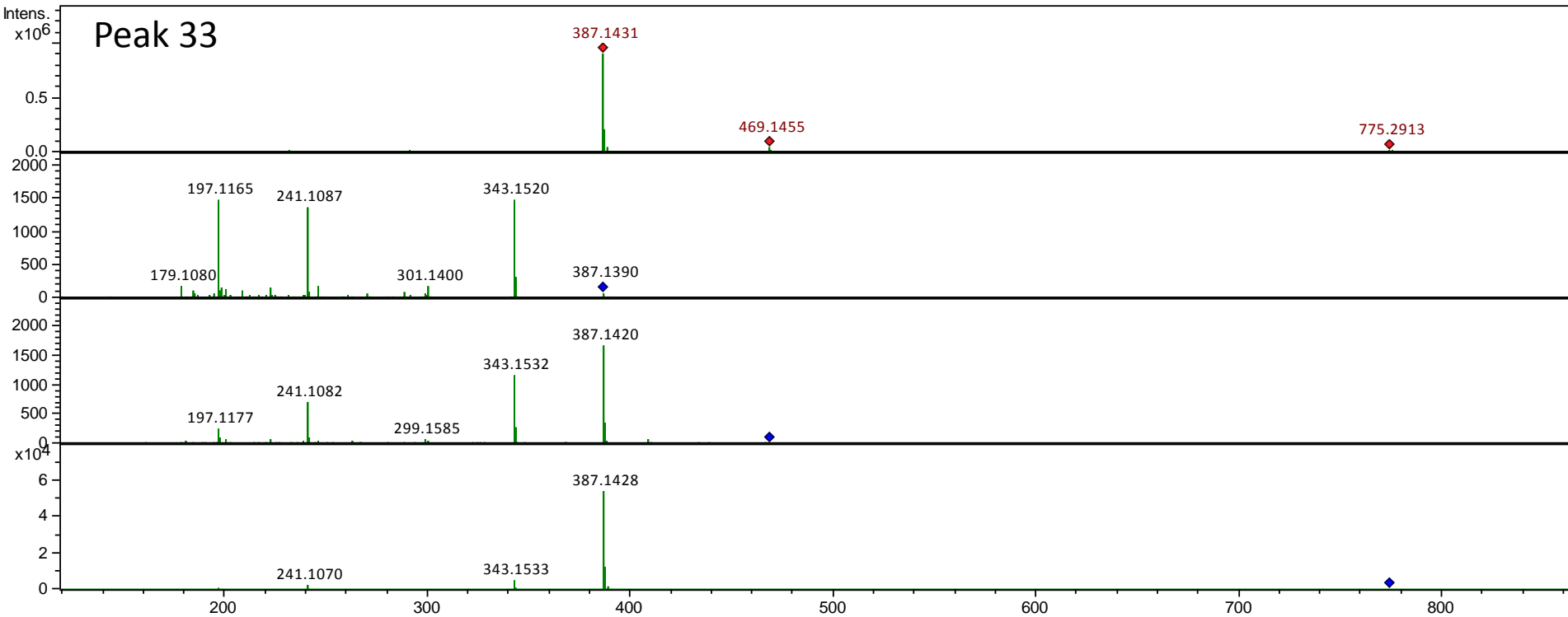

Supplement: S2 Fig — (PDF) [file pone.0211237.s002.pdf]
