# Supplementary material for: Discovery of potential ovicidal natural products using metabolomics
Source: PLoS One. 2019 Jan 25;14(1):e0211237. doi: 10.1371/journal.pone.0211237 (PMC6347362; doi:10.1371/journal.pone.0211237)
Supplement: S1 Table — *[M-2H]-2. (PDF) [file pone.0211237.s003.pdf]

S1 Table. Retention time, mass, molecular formula, chemical class of compounds of figure 2 that were also positively correlated to EC_{90} but with a relative intensity of masses lower than 20000.

Peak	Ret (min.)	$[M-H]^-$	Molecular formula	Class	Compounds
Unknown 11	1.23	173.0414	---	---	Unknown
Unknown 12	1.50	290.0878	---	---	Unknown
Unknown 13	2.34	345.1222	$C_{15}H_{22}O_9$	---	Unknown
Unknown 14	2.49	423.1480	$C_{17}H_{28}O_{12}$	---	Unknown
Unknown 16	3.93	353.0878	$C_{16}H_{18}O_9$	Phenylpropanoid	Caffeoylquinic acid
Unknown 18	10.01	389.1042	$C_{16}H_{22}O_{11}$	---	Unknown
Unknown 19	10.50	473.1292	$C_{20}H_{26}O_{13}$	---	Unknown
Unknown 20	11.27	381.0933	$C_{15}H_{23}ClO_9$	---	Unknown
Unknown 25	16.49	596.1331	$C_{26}H_{28}O_{16}$	Flavonol	Unknown
Unknown 26	16.62	475.0306*	$C_{41}H_{28}O_{27}$	---	Unknown
Unknown 30	17.86	453.0379*	$C_{40}H_{28}O_{25}$	---	Unknown
Unknown 32	18.63	515.1163	$C_{25}H_{24}O_{12}$	Phenylpropanoid	Dicaffeoylquinic acid
Unknown 34	18.80	591.1653	$C_{35}H_{28}O_9$	---	Unknown
Unknown 35	19.00	607.1591	$C_{35}H_{28}O_{10}$	---	Unknown
Unknown 36	19.16	433.0755	$C_{20}H_{18}O_{11}$	---	Unknown
Unknown 42	21.13	507.1467	$C_{24}H_{28}O_{12}$	Phenylpropanoid	Unknown
Unknown 43	23.20	677.1498	$C_{34}H_{30}O_{15}$	---	Unknown
Unknown 44	23.79	677.1491	$C_{34}H_{30}O_{15}$	---	Unknown
Unknown 45	23.95	421.2035	$C_{19}H_{34}O_{10}$	---	Unknown
Unknown 47	30.27	793.9424	$C_{42}H_{66}O_{14}$	---	Unknown
Unknown 53	31.36	558.2535*	$C_{57}H_{82}O_{22}$	---	Unknown
Unknown 55	32.16	939.4924	$C_{48}H_{76}O_{18}$	---	Unknown
Unknown 57	32.85	399.1429	$C_{22}H_{24}O_7$	---	Unknown

$*[M-2H]^{-2}$
